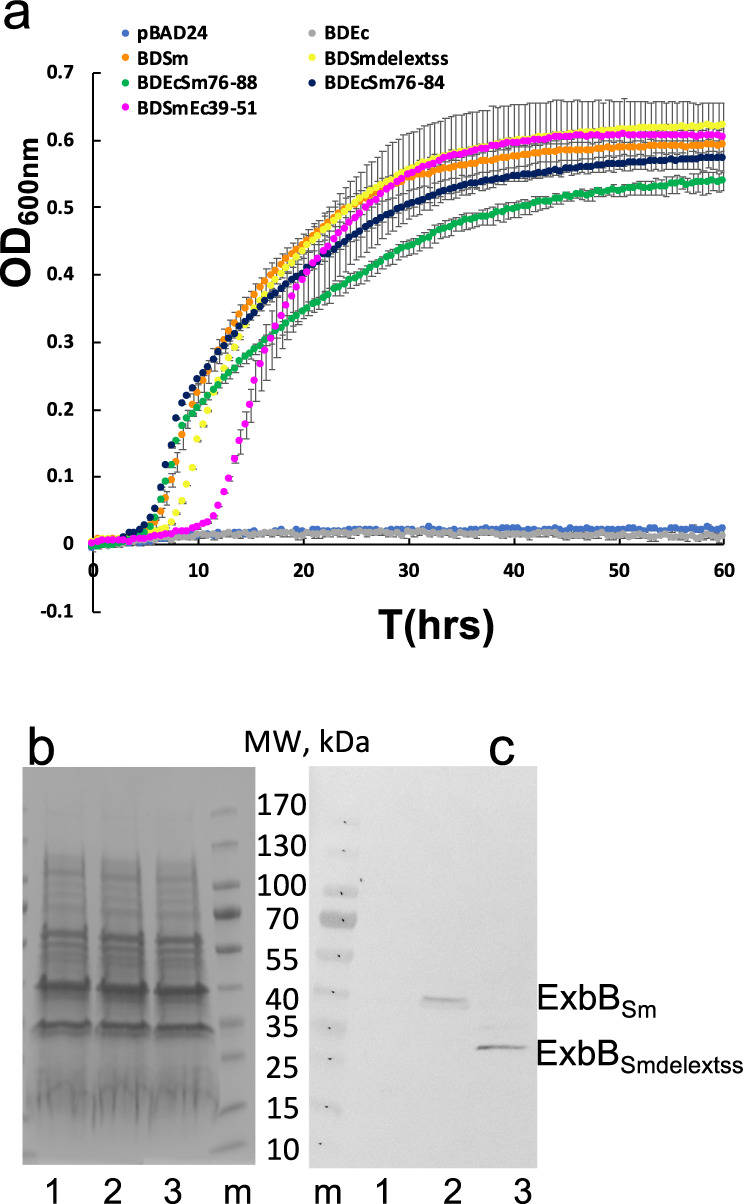# Author Correction: Structural and molecular determinants for the interaction of ExbB from *Serratia marcescens* and HasB, a TonB paralog

**DOI:** 10.1038/s42003-022-04256-1

**Published:** 2023-01-18

**Authors:** Valérie Biou, Ricardo Jorge Diogo Adaixo, Mohamed Chami, Pierre-Damien Coureux, Benoist Laurent, Véronique Yvette Ntsogo Enguéné, Gisele Cardoso de Amorim, Nadia Izadi-Pruneyre, Christian Malosse, Julia Chamot-Rooke, Henning Stahlberg, Philippe Delepelaire

**Affiliations:** 1 Laboratoire de Biologie Physico-Chimique des Protéines Membranaires, Université de Paris, Paris, France; 2 Institut de Biologie Physico-Chimique, Paris, France; 3 Center for Cellular Imaging and NanoAnalytics, Biozentrum, University of Basel, Basel, Switzerland; 4 Laboratoire de Biologie Structurale de la Cellule, BIOC, UMR7654 CNRS/Ecole polytechnique, Palaiseau, France; 5 Plateforme de Bioinformatique, Université de Paris, Paris, France; 6 Structural Bioinformatics Unit, Department of Structural Biology and Chemistry, C3BI, Institut Pasteur, Paris, France; 7 Mass Spectrometry for Biology Unit, CNRS USR 2000, Institut Pasteur, Paris, France; 8 Present Address: Department of Biochemistry, University of Cambridge, Cambridge, UK; 9 Present Address: Instituto de Bioquímica Médica, Universidade Federal do Rio de Janeiro, Rio de Janeiro, RJ Brasil; 10 Present Address: Centre d’imagerie Dubochet UNIL-EPFL-UNIGE & Laboratoire de microscopie électronique biologique UNIL-EPFL, Lausanne, Switzerland

**Keywords:** Bacterial structural biology, Cryoelectron microscopy

Correction to: *Communications Biology* 10.1038/s42003-022-03306-y, published online 13 April 2022.

After the publication of this Article, the authors realised that the incorrect strain had been used for some experiments. Due to a mix-up in the authors’ strain library, the Escherichia coli strain *C600∆tonB∆exbBD∆hemA::Km* believed to be used in was in fact *C600∆exbBD∆hemA::Km* and therefore still contained the *tonB* gene from *E.coli*. The growth experiments shown in Figs. 3b–d and 5a have now been performed with the correct strain. The general outcome of the paper does not change; in particular some discrepancies with previous published findings have now been resolved and no longer exist and the differences seen in the different mutant forms of ExbB are still evident.

The text and figures associated with the new data have been corrected as follows:

On page 4, left column, the original text read:

“The results are reported in Fig. 3b (Petri dishes, overnight observation), 3c (Petri dishes, 36 h observation), and 3D (microplates, recording over 66 h). As expected, control strains (with pBAD24) did not grow. ExbBDSm was functional with both HasB and TonB (middle series of holes in Fig. 3c indicated with orange and blue dots, and orange and blue dots curves in Fig. 3d, Supplementary Data 1), although with quite dramatically different kinetics and yield, the onset of growth occurring at 5–6 h for the HasB-ExbBDSm pair, and at 22–23 h for the TonB-ExbBDSm pair. Moreover, the final OD of TonB-ExbBDSm is half that of HasB-ExbBDSm. Under our experimental setup, ExbBDEc was also functional with both TonB and more surprisingly with HasB (bottom series of holes in Fig. 3c, and green and gray dots curves in Fig. 3d, Supplementary Data 1), the onset of growth occurred at around 15–16 h for TonB, and 18 h for HasB; however, the maximal OD was lower for the HasB-ExbBDEc pair (0.43 vs 0.65). Finally, the ExbBDSm-HasB pair seems less sensitive to iron starvation than all the other ones, since similar results are obtained at 100 and 200 µM Dipyridyl (Fig. 3b), which is not the case for the other pairs, as already observed in the TonB-ExbBDEc case25. These results might seem at odds with the previous results obtained on HasB12 which showed that HasB was nonfunctional with ExbBDEc. However, the experimental setup was quite different, both in terms of plasmids used, strains and conditions of observation.”

The text has been changed to:

“The results are reported in Fig. 3b (Petri dishes, overnight observation), 3c (Petri dishes, 36h observation) and 3d (microplates, recording for 60h). As expected, both in plates and in liquid cultures, control strains (with pBAD24) did not grow (yellow and deep blue in Fig. 3b–d). ExbBD_Sm_ was functional with both HasB and TonB (middle series of holes in Fig. 3b, c indicated with orange and blue dots, and orange and blue dots curves in Fig. 3d, Supplementary Data 1), although with quite dramatically different kinetics, the onset of growth occurring at 3–4 h for the HasB-ExbBD_Sm_ pair, and ca. 20 h for the TonB-ExbBD_Sm_ pair. As previously observed^12^, ExbBD_Ec_ was also functional with TonB (onset of growth at ca. 15 h) but not with HasB (bottom series of holes in Fig. 3b, c, and green and light-grey dots curves in Fig. 3d). The C600*∆hemA∆exbBD∆tonB* strain is more sensitive to iron starvation than the C600*∆hemA∆exbBD* one, as it has no means of acquiring iron (due to the lack of both *tonB* and *exbBD* and as *hasB* does not complement *tonB* for iron acquisition). A lower dipyridyl concentration is therefore required to achieve comparable iron starvation. This is clear in the central part of Fig. 3b or c, where in the presence of ExbBDSm, growth around the wells is observed at 100 µM dipyridyl but hardly at 200 µM in the C600*∆hemA∆tonB∆exbBD*(pAMhasISRADEB) case, the reverse being true in the C600*∆hemA∆exbBD*(pAMhasISRADE) case (orange vs. pale-blue dots). In liquid cultures in microplates, a lower dipyridyl concentration is required to achieve similar iron starvation as in plates. This is why we used 100 µM for C600*∆hemA∆exbBD*(pAMHasISRADE) derivatives and 40 µM for C600*∆hemA∆exbBD∆tonB*(pAMHasISRADEB) derivatives.”

On page 4, at the end of the right column, the following sentence has been deleted:

“In addition, the maximum OD reached after growth is slightly but reproducibly decreased (Fig. 5a, Supplementary Data 2).”

On page 7, the text in the right column read:

However, our data rather favor a less efficient interaction between HasB and ExbBD_Ec_ than with ExbBD_Sm_. *E. coli* can grow with the HasB-ExbBD_Ec_ pair_,_ although more slowly and with a lower yield: the final OD was 0.78 for HasB-ExbBD_Sm_ and 0.43 for HasB-ExbBD_Ec_ (Fig. 3d orange and grey curves).

The text has been changed to:

However, our data rather favor a non-functional interaction between HasB and ExbBD_Ec._

At the end of the paragraph, the text stated:

This observation therefore indicates that most likely HasB also interacts with ExbBD_Ec_ so as to be stabilized (i. e., withstanding proteolytic degradation), but in a less functional manner than with ExbBD_Sm_.

The text has been changed to:

“This observation therefore indicates that most likely HasB also interacts with ExbBD_Ec_ so as to be stabilized (i. e., withstanding proteolytic degradation), but in a non-functional manner.”

The final paragraph on page 7 read:

“As shown in Fig. 5a, this chimeric protein ExbBEc-Sm76-88 (green dots) was more active with HasB than ExbBEc (gray), the onset of growth took 14 h compared to 18 h for ExbBDEc and the maximum OD was 0.74 compared to 0.43 for ExbBDEc. We also made the inverse change where the 76–88 region of ExbBSm was replaced by the 39–51 region from ExbBEc (ExbBSm-Ec39–51, magenta). This mutant has a degraded behavior as compared to ExbBSm, since the onset of growth occurs at 19 h …. ”

The text has been changed to:

“In this last case, as already seen, there is no growth in the presence of HasB and ExbBD_Ec_ (Fig. 5a, grey curve), whereas the growth in the presence of ExbBD_Ec-Sm76-88_ and HasB is indistinguishable (or even with a quicker onset) from the one with ExbBD_Sm_ and HasB (Fig. 5a, green curve, compared to orange curve).”

In the final paragraph of the Results section, the following sentence has been added after “…likely govern the interaction between ExbB and HasB.”:

“The substitution, in ExbB_Sm_ TM1 helix of the corresponding residues from ExbB_Ec_ (ExbBD_SmEc39-51_), degraded the activity of ExbB_Sm_, with a longer lag before onset of growth (10 vs. 3-4 hrs, orange and magenta curves).”

The following sentences were added to the Discussion after “…transcription activation of the Has locus.”:

“In the context of the live bacterium, where both *tonB* and *hasB* are present, the existence of the various potential interactions between ExbBD and TonB or HasB might allow to fine-tune the use of the various iron acquisition systems to the surrounding iron sources. This shows the promiscuity of the ExbBD system from *S. marcescens*, and one might speculate that dedicated ExbBD systems will coexist in a given bacterium with more promiscuous ones.”

In the Methods, Activity test point 3, the text read:

“Growth curves in liquid medium: a few colonies of E. coli C600 *ΔhemAΔtonBΔexbBD*(pAMHasISRADEB + pBAD24 or derivatives thereof) were first inoculated in 4 ml of LB medium at 37 °C with the corresponding antibiotics, and 100 µM dipyridyl, 4 µg/ml arabinose but without delta-aminolevulinic acid.”

The text has been changed to:

“Growth curves in liquid medium: a few colonies of *E. coli* C600 *∆hemA∆tonB∆exbBD*(pAMHasISRADEB + pBAD24 or derivatives thereof) or C600*∆hemA∆exbBD*(pAMHasISRADE + pBAD24 or derivatives thereof) were first inoculated in 4ml of LB medium at 37°C with the corresponding antibiotics, and 40 µM or 100µM dipyridyl respectively, 4 µg/ml arabinose but without delta-aminolevulinic acid.”

The legend to figure 3 said:

“DiP concentration was 100 µM,…”

The text has been changed to:

“DiP concentration was 100 µM in the C600*∆hemA∆exbBD* (pAMHasISRADE) derivatives, and 40 µM for C600*∆hemA∆tonB∆exbBD* (pAMHasISRADEB) derivatives,…”

The legend to figure 5 said:

“and 100 µM dipyridyl to induce iron starvation….”

The text has been changed to:

“and 40µM dipyridyl to induce iron starvation….”

Figure 3 has been replaced:

Previous Figure 3:
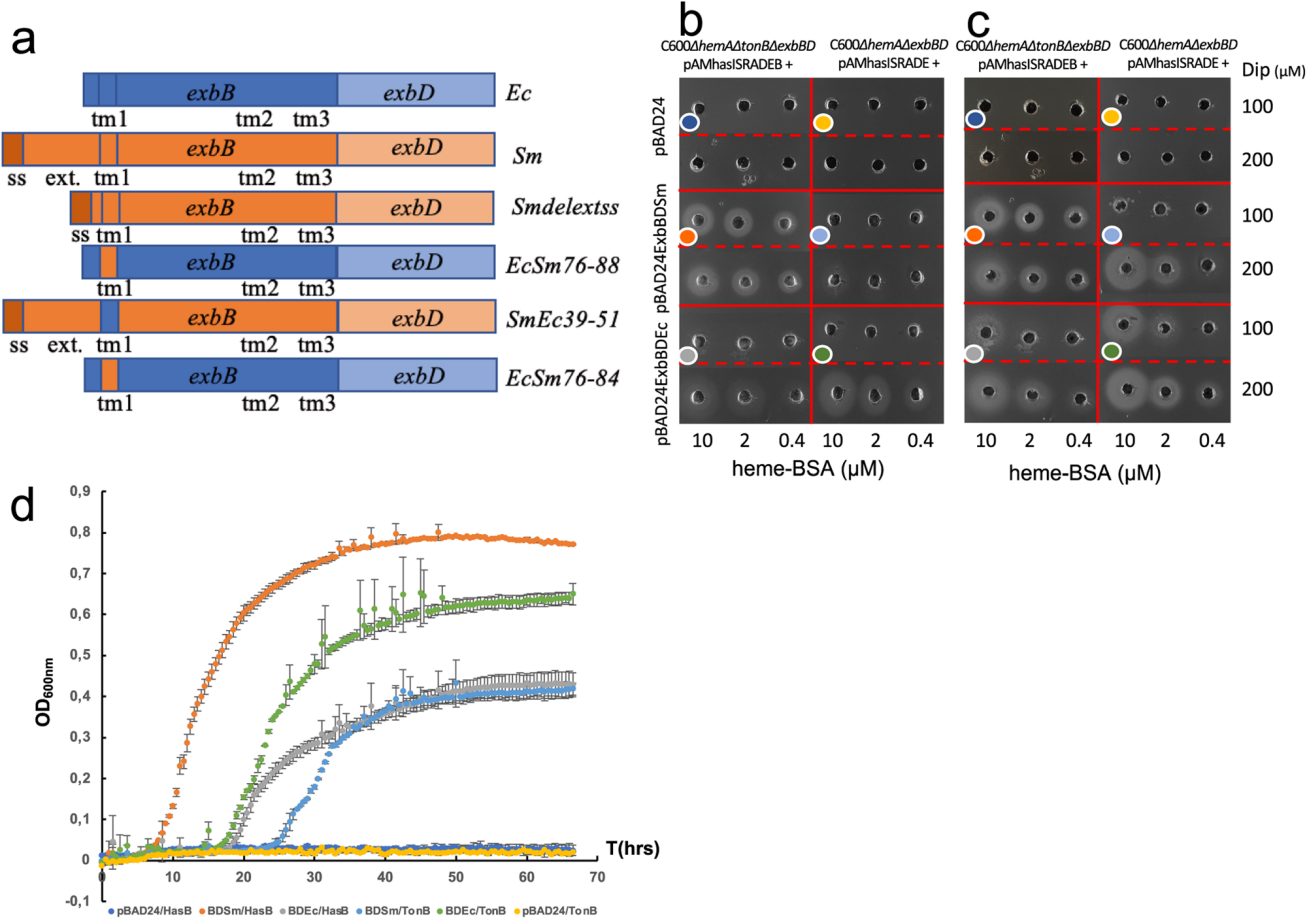


New Figure 3:
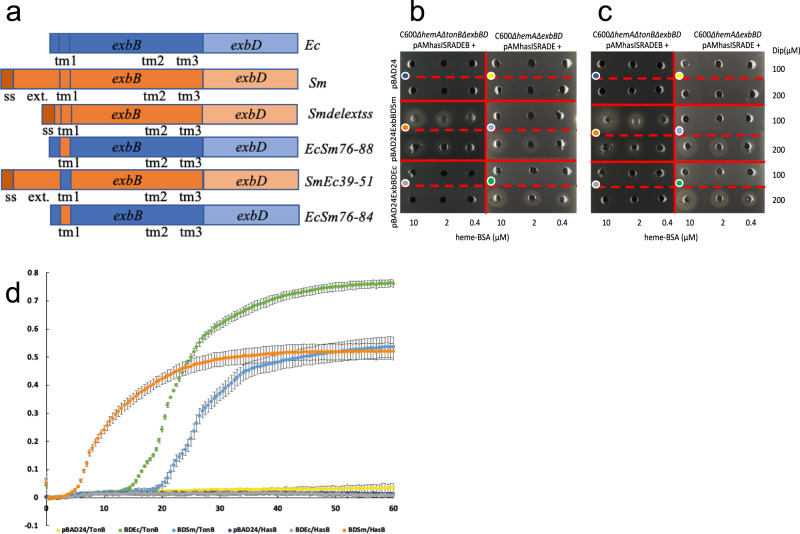


Figure 5a has been replaced:

Previous figure 5
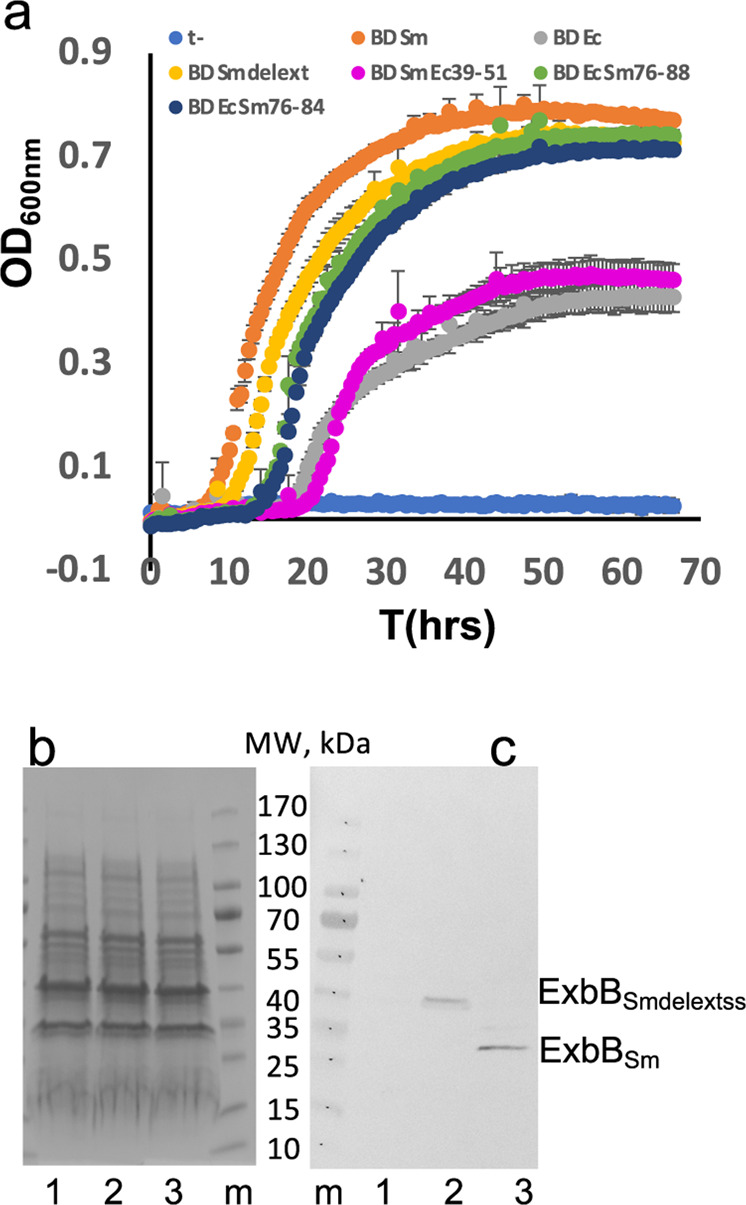


New Figure 5