# Supplementary material for: Structural and molecular determinants for the interaction of ExbB from Serratia marcescens and HasB, a TonB paralog
Source: Commun Biol. 2022 Apr 13;5:355. doi: 10.1038/s42003-022-03306-y (PMC9008036; doi:10.1038/s42003-022-03306-y)
Supplement: Supplementary file 2 — Supplementary Information [file 42003_2022_3306_MOESM2_ESM.pdf]

Structural and molecular determinants for the interaction of ExbB from *Serratia marcescens* and HasB, a TonB paralog.

Valérie Biou#, Ricardo Jorge Diogo Adaixo, Mohamed Chami, Pierre-Damien Coureux, Benoist Laurent, Véronique Yvette Ntsogo Enguéné , Gisele Cardoso de Amorim, Nadia Izadi-Pruneyre, Christian Malosse, Julia Chamot-Rooke, Henning Stahlberg, Philippe Delepelaire#

correspondence valerie.biou@ibpc.fr

correspondence philippe.delepelaire@ibpc.fr

This file contains the following items:

supplementary Figures S1 to S15

supplementary Tables 1 and 2

Other supplementary information include

supplementary Data 1 (separate file) associated to Figure 3D

supplementary Data 2 (separate file) associated to Figure 5A

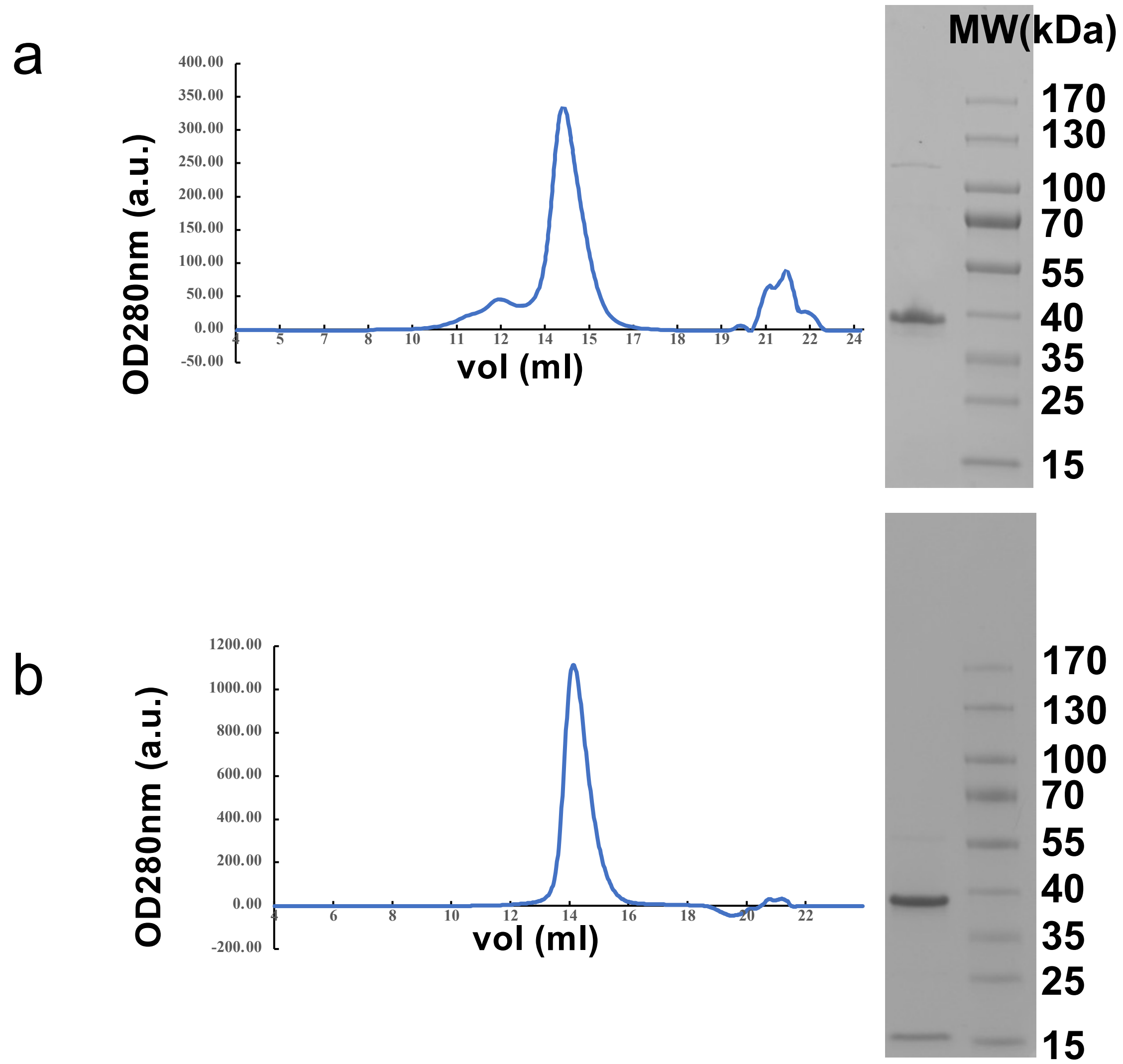

Supplementary Figure S1: Homogeneity assessment of ExbB and ExbBD. Size exclusion chromatography profiles of representative purification of respectively ExbB$_{Sm}$His6 (a) and ExbBD$_{Sm}$His6 (b) on a Superose 6 10/300 column. The 280nm absorbance is plotted as a function of elution volume. A Coomassie-stained gel of the pooled peak fractions is shown on the right of each profile together with a molecular weight ladder on the right (respectively 15, 25, 35, 40, 55, 70, 100, 130 and 170kDa). The faint band present in the Exb B$_{Sm}$His6 sample above 100kDa was identified as AcrB using mass spectrometry.

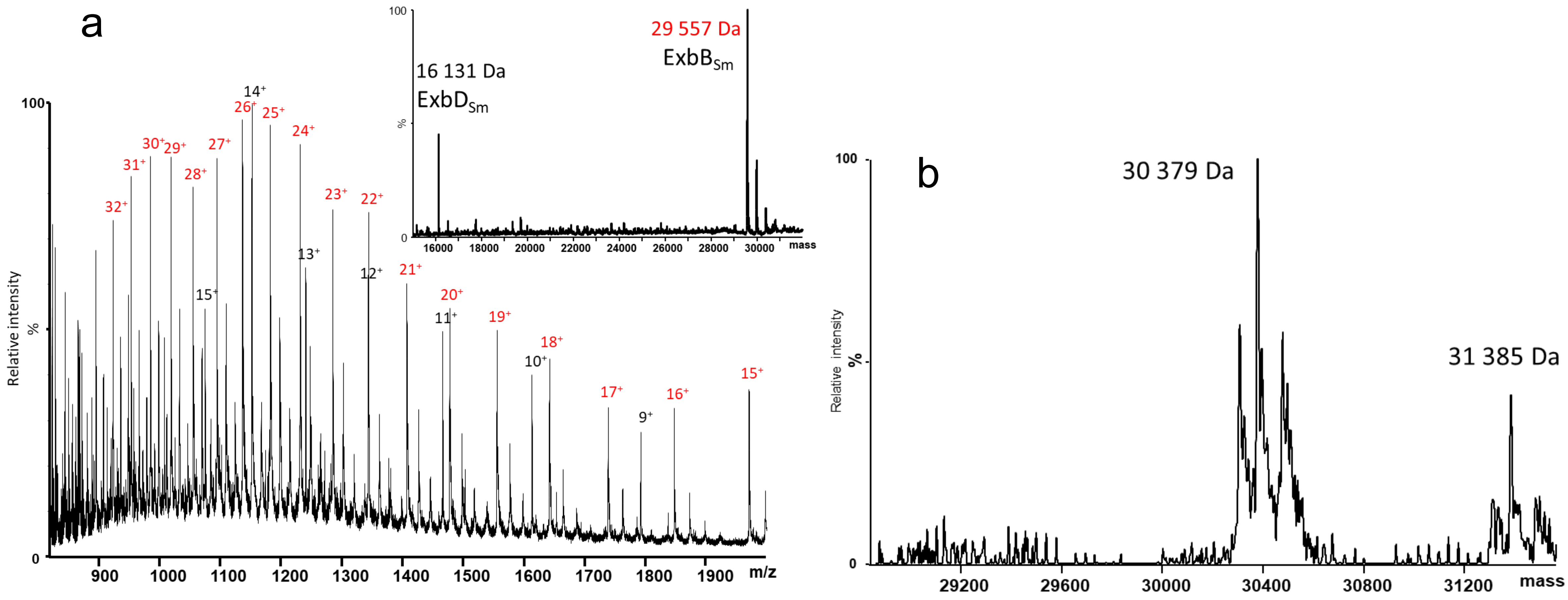

Supplementary Figure S2: Mass spectra of full-length proteins used in this study. a. MS spectrum (deconvoluted data in insert) of the ExbBD$_{Sm}$ complex in denaturing conditions (Mr$_{theoretical}$ ExbB$_{Sm}$ = 29 557 Da after signal peptide removal, Mr$_{theoretical}$ ExbD$_{Sm}$ = 16 162 Da after initial Met removal). b. Deconvoluted mass spectrum of purified ExbB$_{Sm}$ (Mr$_{theoretical}$ = 30 380 Da) showing the presence of the protein and an adduct with LMNG (Mr$_{theoretical}$ ExbB$_{Sm}$ + LMNG =31 385 Da).

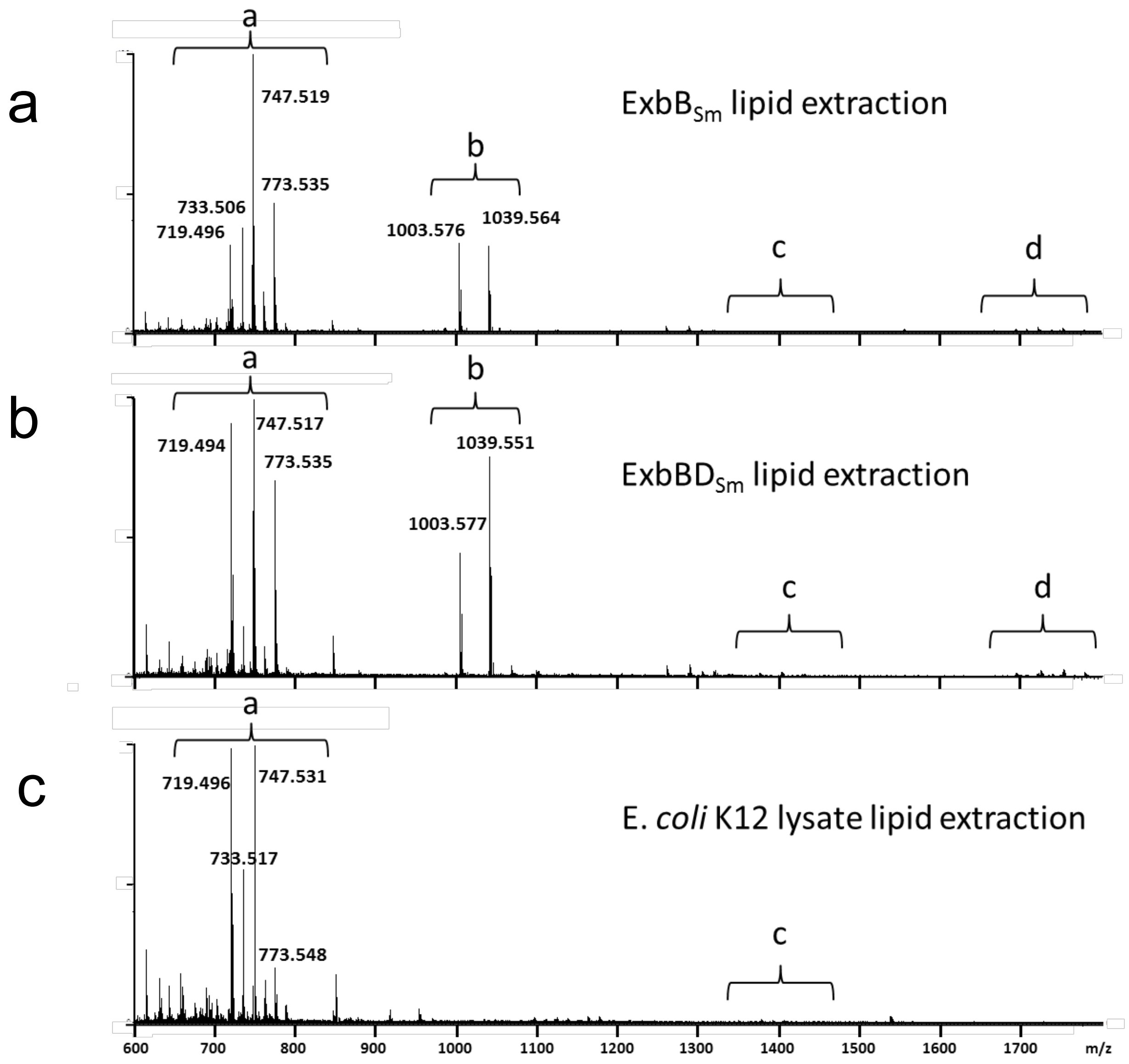

Supplementary Figure S3: Mass spectrometry analysis of lipid extracts for the following samples: a. ExbB$_{Sm}$ b. ExbBD$_{Sm}$ and $_{c}$. *E. coli* lysate. The (a) area corresponds to PE (phosphatidylethanolamine) and PG (Phosphatidylglycerol) derivatives; (b) to LMNG; (c) to cardiolipins and (d) to PE/PG LMNG adducts.

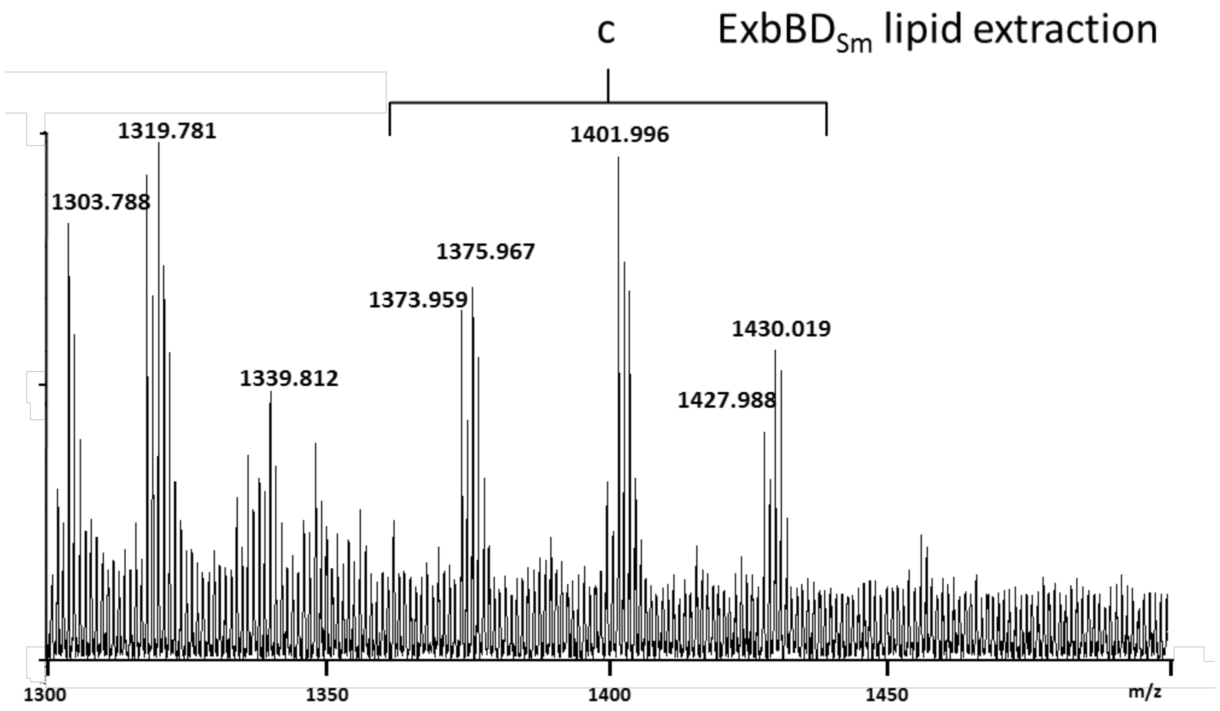

Supplementary Figure S4: Zoom of Figure S3B on the (c) area showing the different cardiolipins.

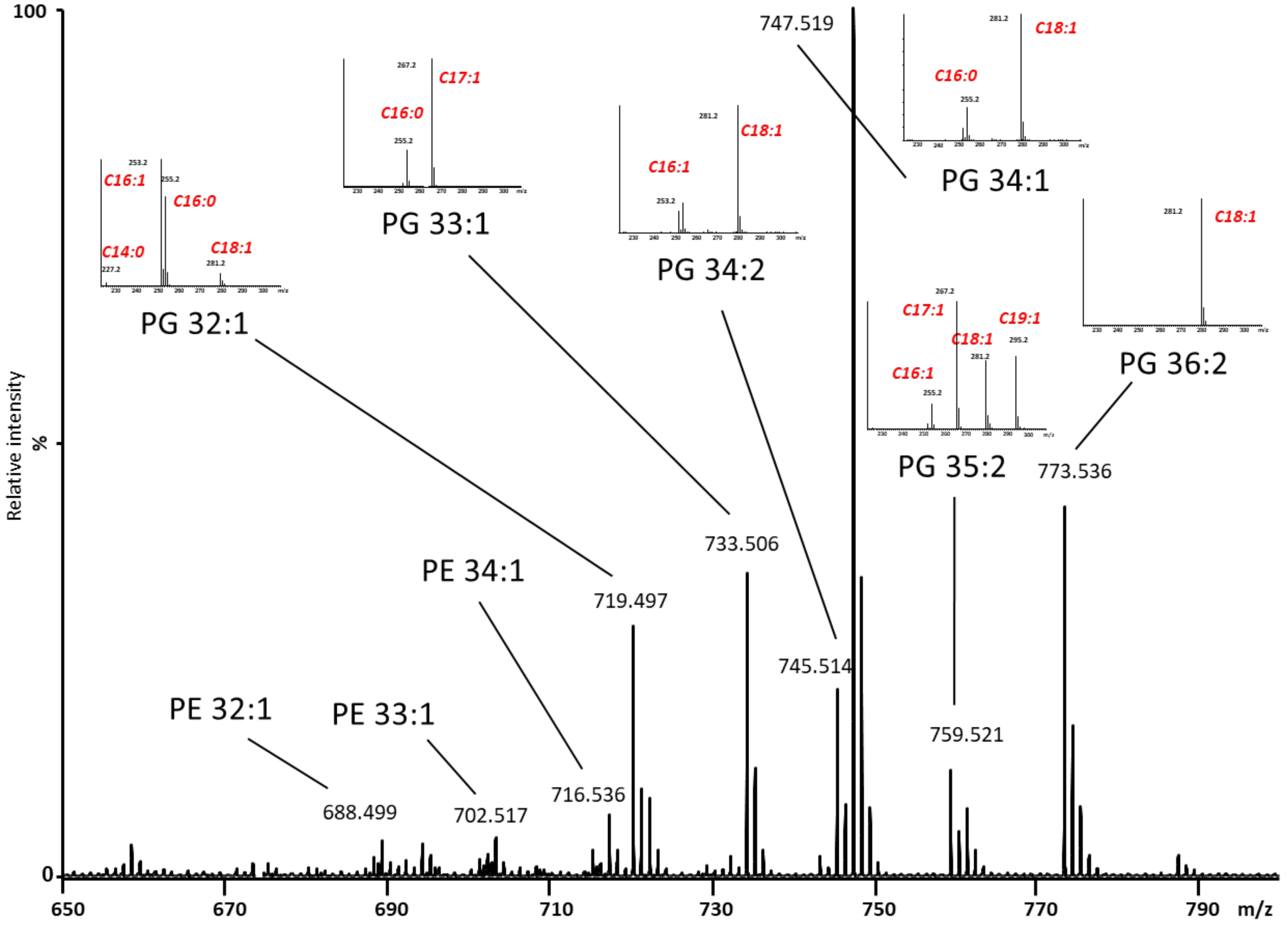

Supplementary Figure S5: Zoom of Figure S3A on the (a) area including CID spectra of the most intense ions in inserts. PE: phosphatidylethanolamine, PG: phosphatidylglycerol, the number of carbons and insaturations are also indicated.

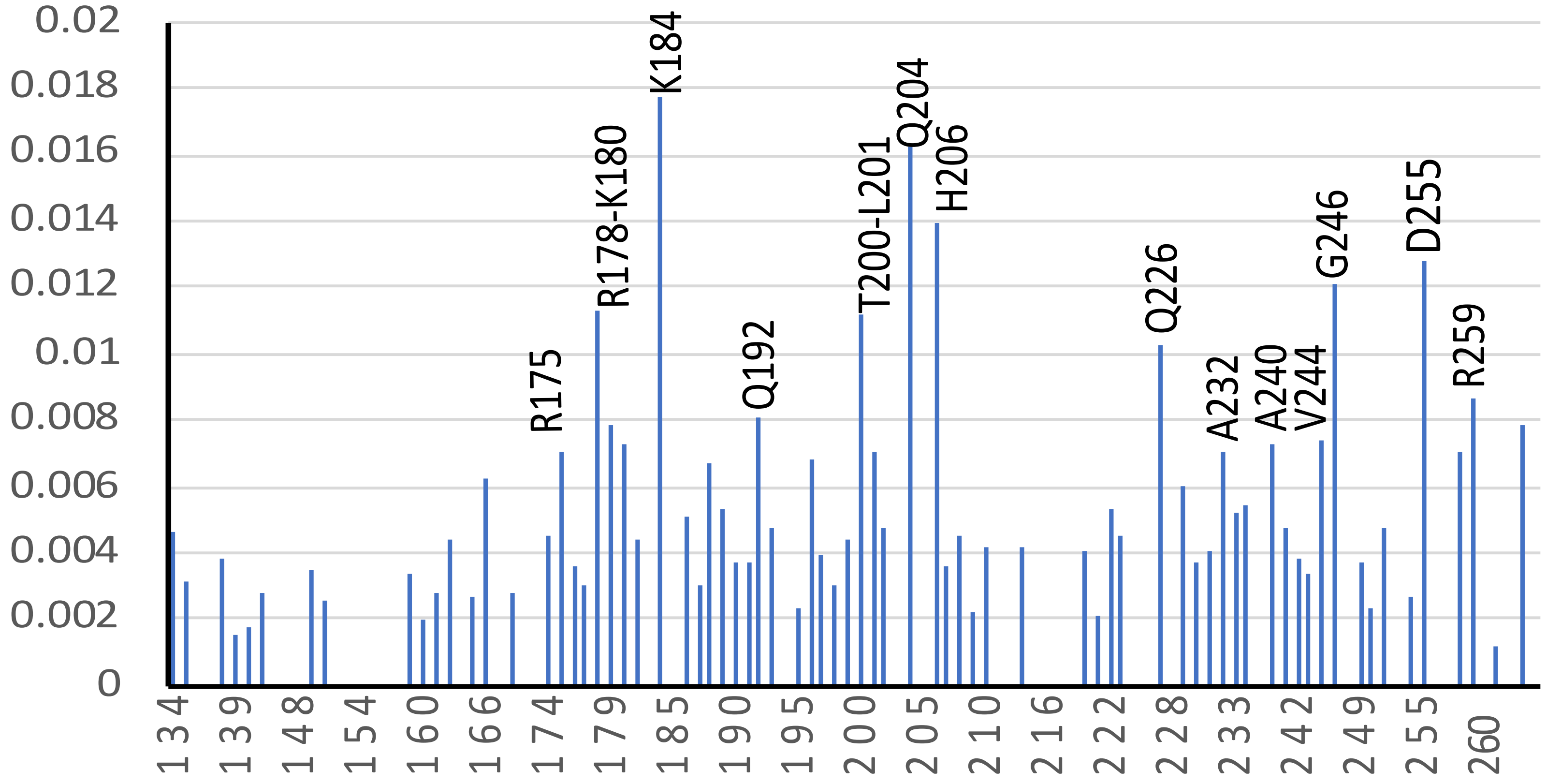

Supplementary Fig S6: Histogram showing the chemical shift perturbation (CSP) values of backbone amide signals of $HasB_{CTD}$ (0.15 mM in 50mM sodium phosphate, pH 7, 50 mM NaCl) in the presence of the $ExbB_{Sm}$ 1-44 peptide, as a function of residue numbers. The protein/peptide ratio was 1:10. Residues showing CSP higher than 0.007 are considered for analysis, except the last residue.

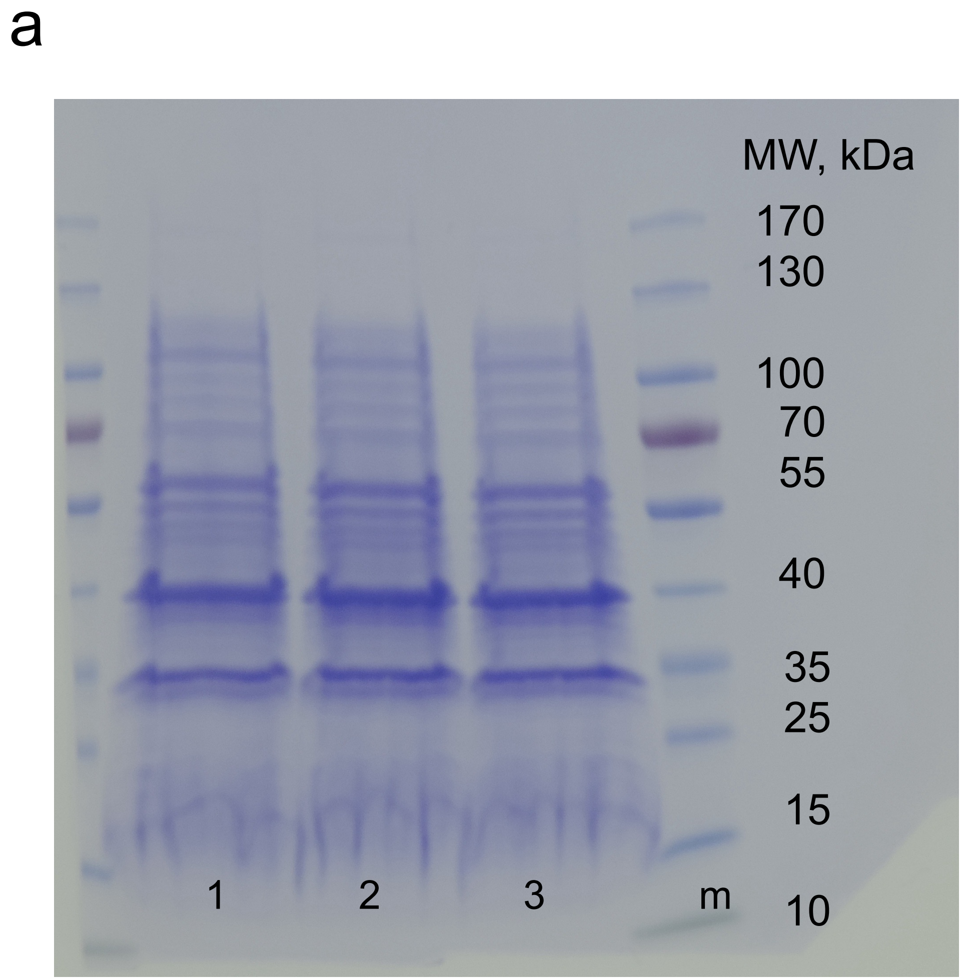

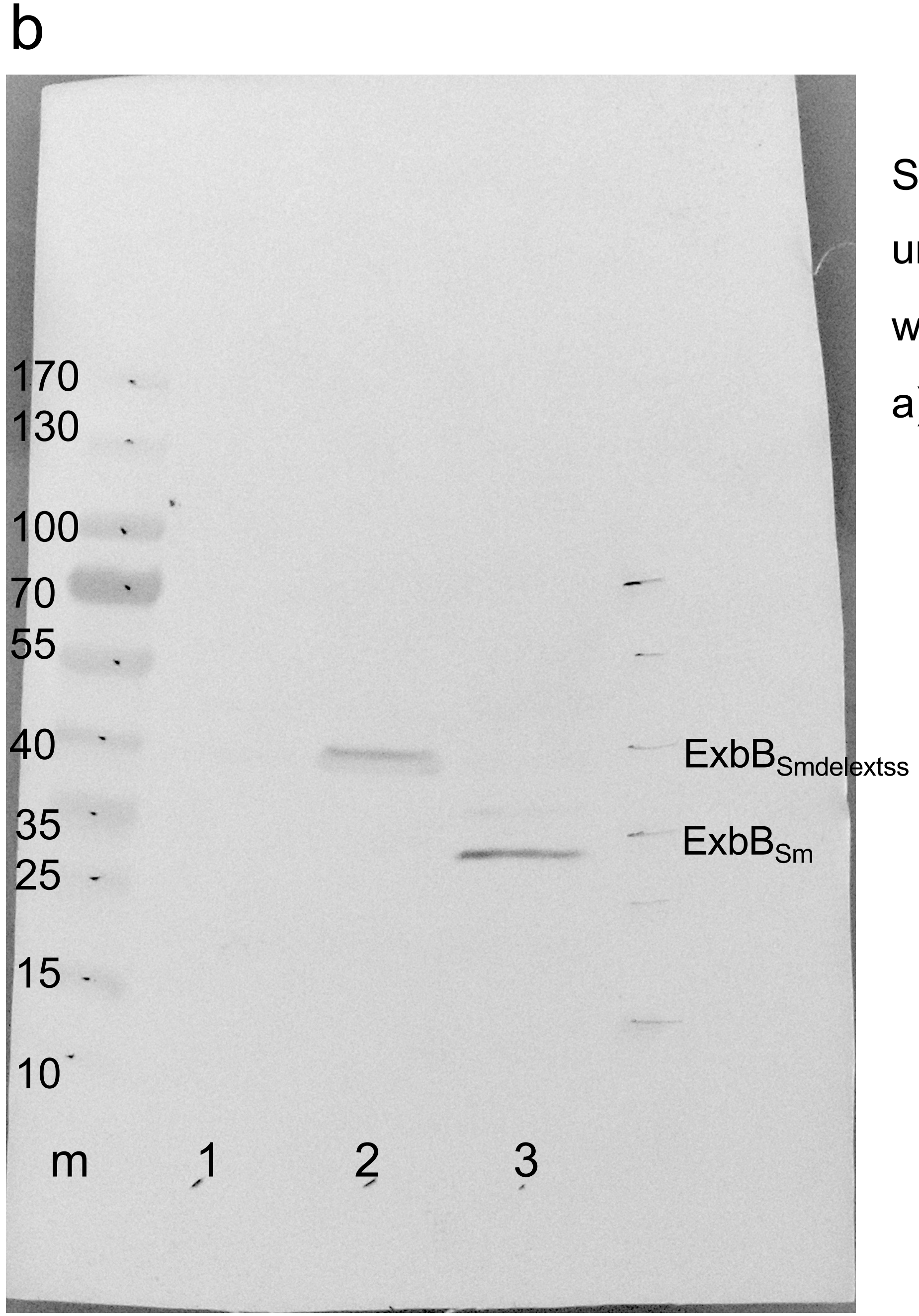

Supplementary Figure S7: uncropped SDS-PAGE and western blot corresponding to a) Figure 5b and b) Figure 5c.

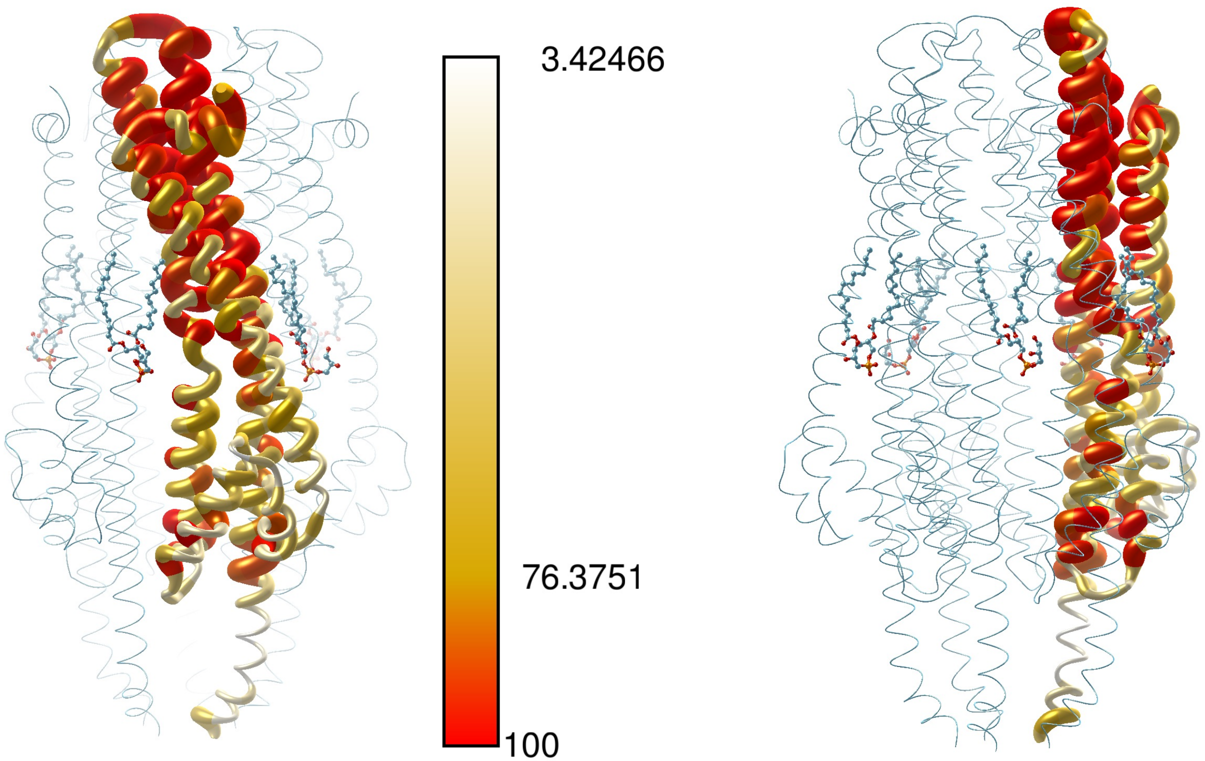

Supplementary Figure S8: sequence conservation in ExbB. structure of ExbB with one monomer ramp-colored with respect to sequence conservation using sequences retrieved in Table SI and Consurf server. Worm diameter also increases with conservation. In the transmembrane region the TM1 is the least conserved.

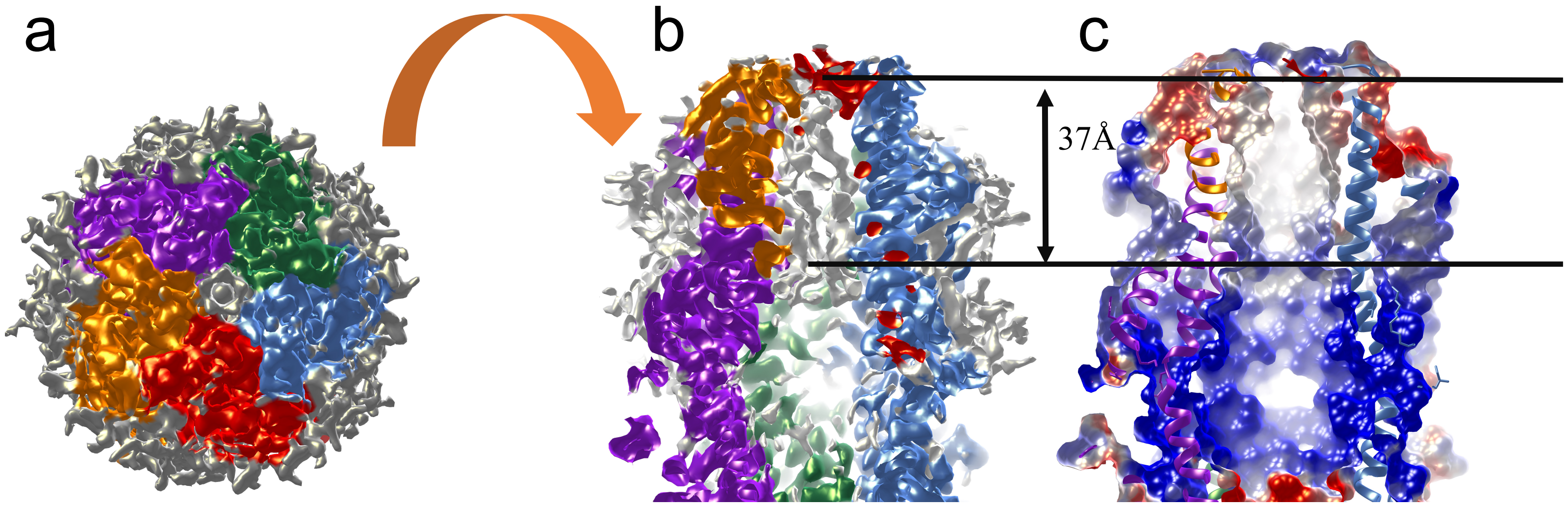

Supplementary figure S9: ExbB pentamer hosts density inside its transmembrane channel. a and b, top and side views of the ExbB density colored with respect to protein chains as in Figure 8. the grey regions show extra density not accounted for by the model. c, electrostatic surface inside the channel corresponding to b. The two black lines show the cytoplasmic and periplasmic limits of the channel density. They are 37Å away from each other.

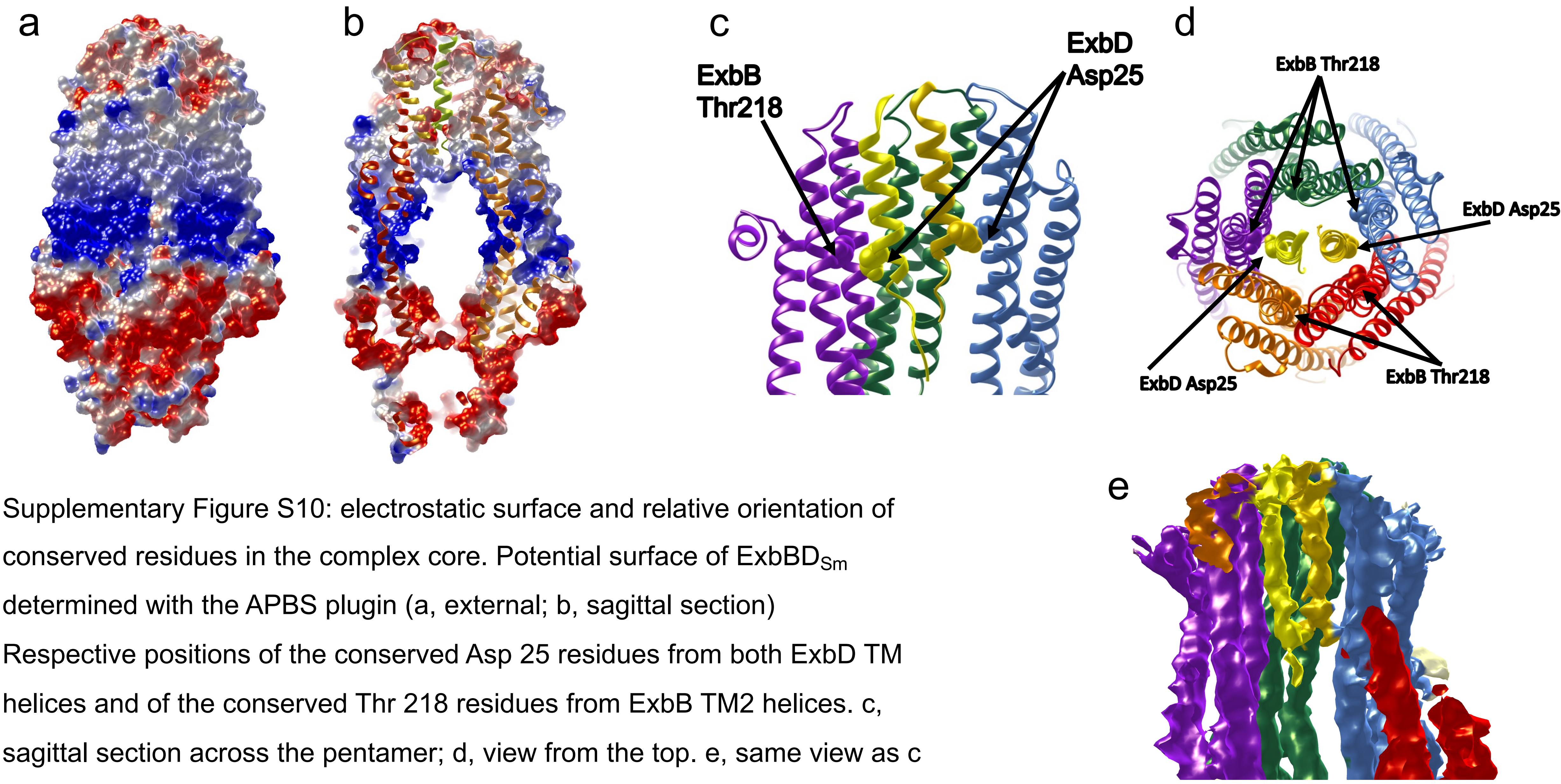

Supplementary Figure S10: electrostatic surface and relative orientation of conserved residues in the complex core. Potential surface of $ExbBD_{Sm}$ determined with the APBS plugin (a, external; b, sagittal section) Respective positions of the conserved Asp 25 residues from both ExbD TM helices and of the conserved Thr 218 residues from ExbB TM2 helices. c, sagittal section across the pentamer; d, view from the top. e, same view as c with the density map.

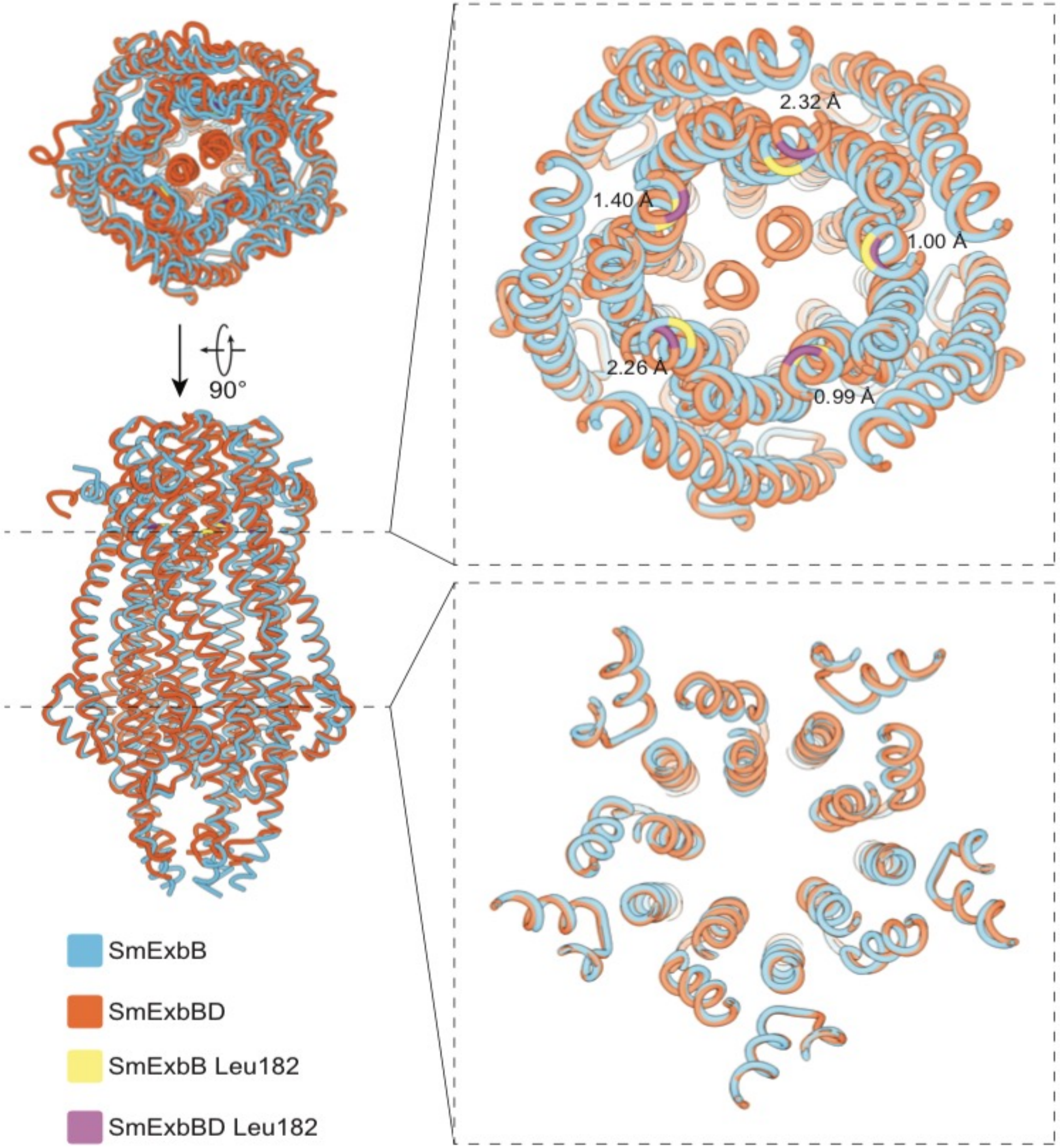

Supplementary Figure S11. Comparison of ExbBSm with ExbBDSm (top inset: section view from the periplasmic side, bottom inset: section view from the cytoplasmic side): both ExbB structures are represented in cartoon (ExbB, lightblue colour, ExbBD lightbrown colour, respectively). A specific Leu 182 residue at the periplasmic entrance is highlighted with the Cα distances between the two molecules.

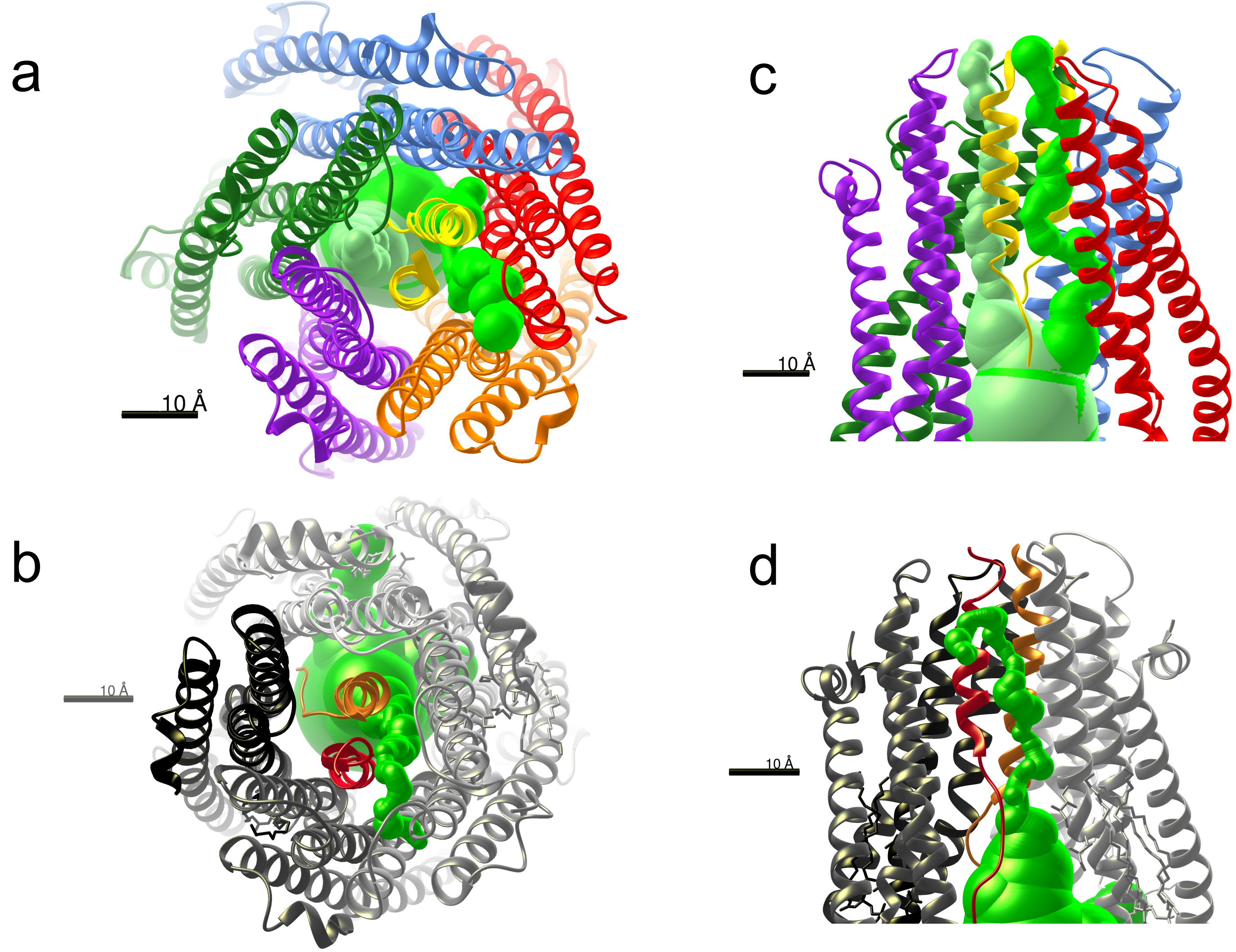

Figure S12: Visualisation of “tunnels”. ExbBD structures from *S. marcescens* (a and c) and *E. coli* 6TYI structure (b and d), as calculated by the MoleOnline server with the same parameters. The Sm complex is colored as in Figure 8 and the Ec complex is coloured in shades of grey for ExbB and orange and red for ExbD. The tunnels are the green volumes running through the structure, viewed from the periplasmic space (a and b), and from the side (c and d). The average tunnel diameter is 3Å for *S. marcescens* and 2Å for *E. coli.*

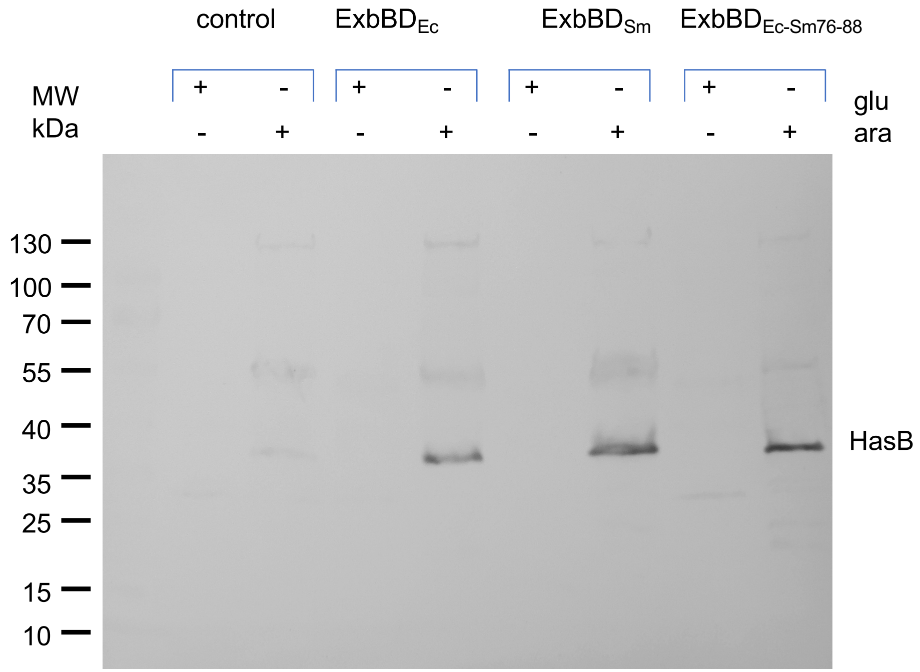

Supplementary Figure S13. ExbBD co-expression is necessary for HasB stabilisation. Immunodetection of HasB in whole cells of *E. coli* JP313Δ*exbBD*Δ*tonB*(pHasB33) also harbouring pBAD24 (control), pBAD24ExbBD$_{Ec}$, pBAD24ExbBD$_{Sm}$ or pBAD24ExbBD$_{Ec\text{-}Sm76\text{-}88}$, in the presence of either glucose (1mg/ml) or arabinose (40µg/ml), indicated by the + signs. The equivalent of 0.2OD$_{600nm}$ was loaded in each lane. The molecular weight markers were located using a more saturated version of this image and are figured by lanes.

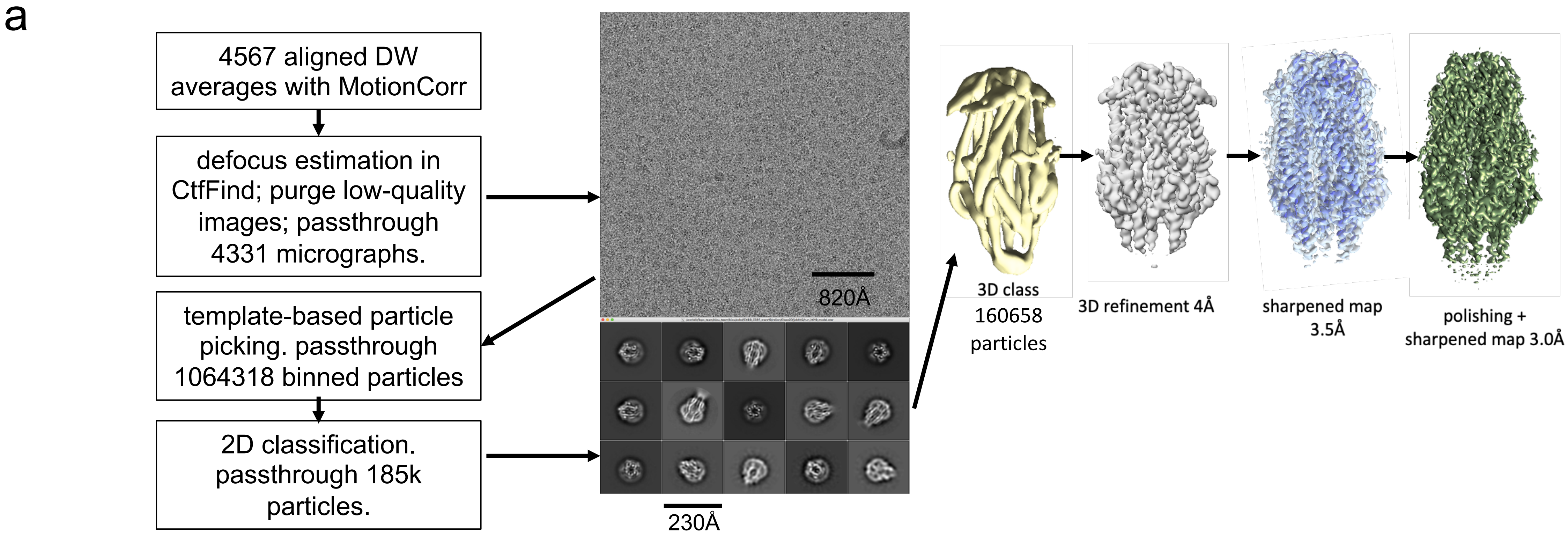

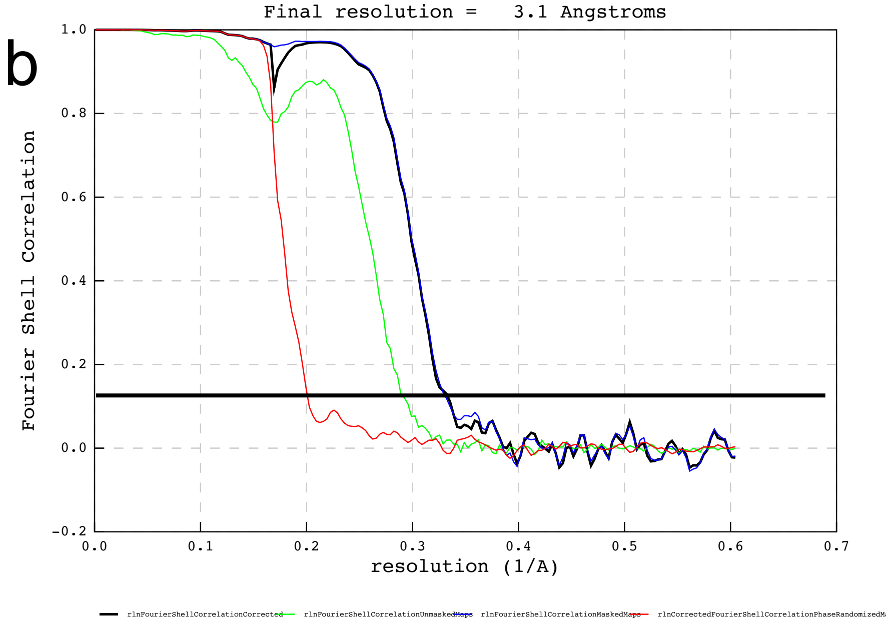

Supplementary Figure S14: Processing of ExbB cryo-EM data set (a) and final Fourier Shell Correlation plot (b).

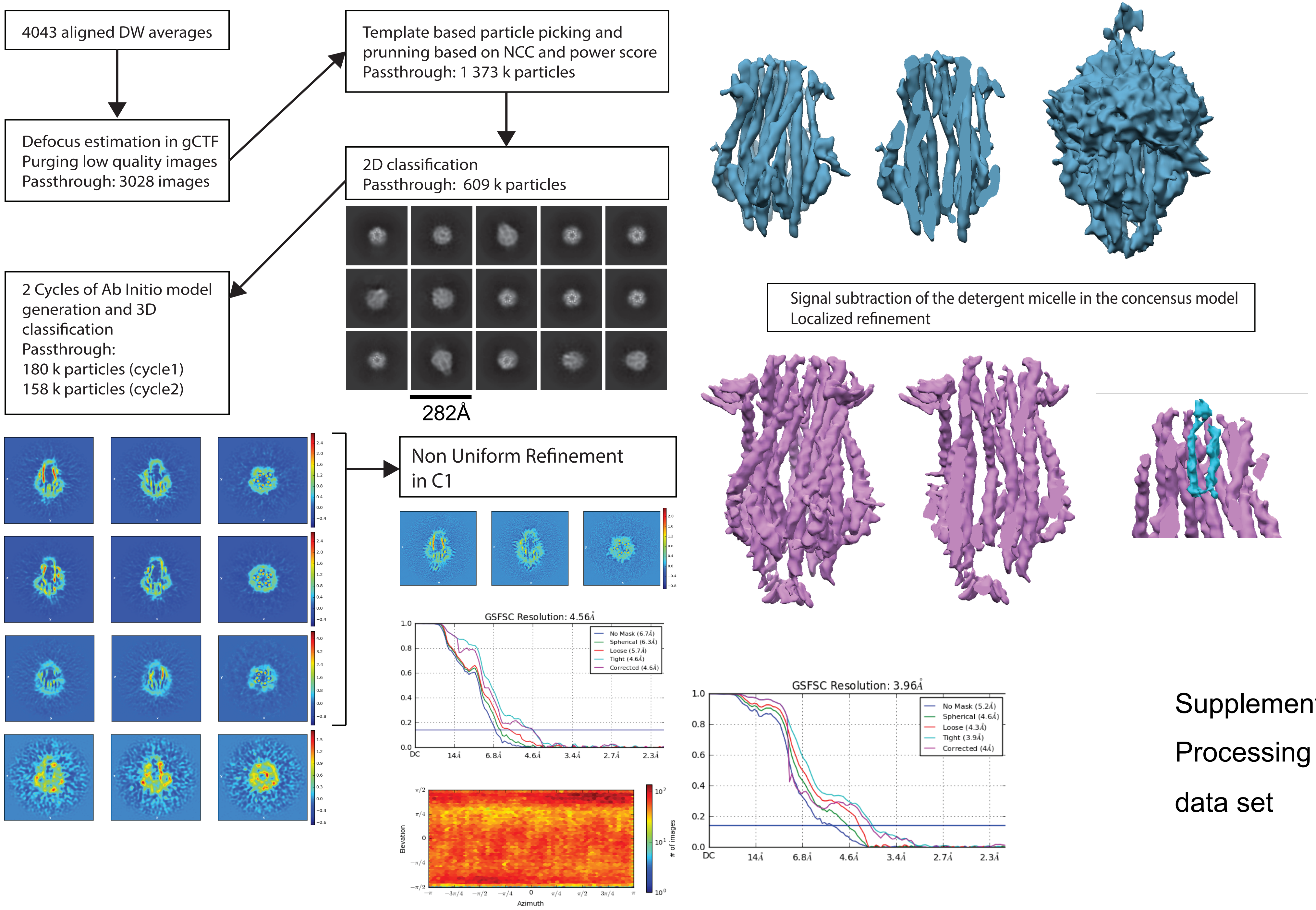

Supplementary Figure S15:

Processing of ExbB-D cryo-EM

data set

Afipia broomeae	Hartmannibacter diazotrophicus	Phreatobacter stygius
Agrobacterium rhizogenes	Hoeflea olei	Phyllobacterium zundukense
Aliihoeflea sp.	Hyphomicrobium nitrativorans	Proteobacteria bacterium
Aminobacter aminovorans	Insolitispirillum peregrinum	Pseudaminobacter manganicus
Amorphus coralli	Kaistia granuli	Pseudochrobactrum asaccharolyticum
Ancylobacter aquaticus	Ketogulonicigenium robustum	Pseudolabrys taiwanensis
Aquamicrobium aerolatum	Labrys okinawensis	Pseudorhizobium pelagicum
Aurantimonas manganoxydans	Magnetospirillum gryphiswaldense	Pseudorhodoplanes sinuspersici
Aureimonas flava	Mangrovicella endophytica	Reyranella massiliensis
Azorhizobium caulinodans	Mesorhizobium loti	Rhizobium tropici
Azospirillum brasilense	Methylobrevis pamukkalensis	Rhodoligotrophos sp.
bacterium A52C2	Methylocapsa acidiphila	Rhodomicrobium udaipurense
Bartonella apis	Methyloceanibacter stevinii	Rhodopseudomonas pseudopalustris
Beijerinckiaceae bacterium	Methylocella silvestris	Rhodospirillum centenum
Blastochloris sp.	Methyloligella halotolerans	Rhodovarius sp.
Bosea vaviloviae	Methylopila sp.	Roseococcus sp.
Bradyrhizobium yuanmingense	Methylovirgula ligni	Roseospirillum parvum
Brucella sp.	Microvirga aerophila	Sinirhodobacter sp.
Chelatococcus asaccharovorans	Neorhizobium alkalisoli	Sinorhizobium sp.
Ciceribacter lividus	Nitratireductor pacificus	Skermanella aerolata
Devosia psychrophila	Nitrobacter hamburgensis	Sphingobium chlorophenolicum
Dongia mobilis	Nitrospirillum amazonense	Sphingomonas sp.
Enterovirga rhinocerotis	Niveispirillum cyanobacteriorum	Sphingopyxis sp.
Erythrobacter	Ochrobactrum thiophenivorans	Sphingorhabdus sp.
Falsochrobactrum ovis	Paenirhodobacter enshiensis	Starkeya novella
Geminicoccus roseus	Paracoccus aminovorans	Tardiphaga robiniae
Granulibacter bethesdensis	Paramesorhizobium deserti	Telmatospirillum siberiense
Haematobacter massiliensis	Parvibaculum lavamentivorans	Variibacter gotjawalensis
Hansschlegelia zhihuaiae	Phaeospirillum fulvum	Xanthobacter autotrophicus

Table S1: a list of selected species of Alphaproteobacteria where “extralong” ExbB’s are found. All those species also contain HasB orthologs, except for the ones in red.

Strains :

E. coli K12 C600
E. coli K12 C600*ΔhemA::Km*^{r}*ΔexbBD*
E. coli K12 C600*ΔhemA::Km*^{r}*ΔexbBDΔtonB*
E. coli K12 XL1-Blue
E. coli K12 JP313
E. coli K12 JP313*ΔexbBDΔtonB*
E. coli K12 C600*ΔexbBD*
E. coli BL21DE3
S. marcescens Db11

Plasmids :

pBAD24: lab collection
pBAD33: lab collection
pAM238: lab collection
pAMHasISRADE: lab collection
pAMHasISRADEB: this work
pBADExbBD$_{Ec}$: this work
pBADExbBD$_{Sm}$: this work
pBADExbBD$_{Ec-Sm76-88}$: this work
pBADExbBD$_{Ec-Sm76-84}$: this work
pBADExbBD$_{Sm-Ec39-51}$: this work
pBADExbBD$_{delextss}$: this work
pBADHasB: lab collection
pBADExbBD$_{Sm}$His6: this work
pBADExbB$_{Sm}$His6: this work
pBADExbB$_{Smdelextss}$His6: this work

Oligonucleotides:

ExbBDSm5′	5′-GGAGGAATTCACCATGAAAACGGCTGGCAAGAAT -3′
ExbBDSm3′	5′-AAGCTTGCATGCCTATTTGGCGGCGCCTTCCA-3′
SphIHisCtexbDSm	5′-TTGCATGCCTAATGGTGATGGTGATGGTGTTTGGCGCGCCTTC-3′
BglIIexbDSm	5′-GCGCTTAAATGAAGATCTGGACGACAGCGG-3′
ExbBD5c	5′-CAGGAGGAATTCACCATGGGTAATAATTTAATGCAGACGGA-3′
ExbBD3c	5′-AAGCTTGCATGCTTACTTCGCTTTGGCGGTTTCTT-3′
PBADFOR	5′-CTGACGCTTTTTATCGCAAC-3′
ExbBHis6	5′-AAGCTTGCATGCCTAATGGTGATGGTGATGGTGCCCCGCCCGCAGTTG-3′
ExbBEcSm76-88.1	5′P-GGCAGTGAACTGCTGCGCGCCAAGCGTCGCCTTAAGCGCGAG-3′
ExbBEcSm76-88.2	5′P-TTTAGCGAACAAAATGGTCCAGGTGACTACGGAGGCCAAAAT-3′
ExbBEcSm76-84.1	5′P-CTAAAGGCAGTGAATTCTTCAATCAGAAGCGTCG-3′
ExbBEcSm76-84.2	5′P-CGAACAAAATGGTCCAGGTGACTACGGAGGCCA-3′
ExbBSmEc39-51.1	5′P-GCGTAGAGTTCTTCAATCAGAAGCGCCGTCTGCGTCGCGA-3′
ExbBSmEc39-51.2	5′P-TCTTACTGAAGAAGATTGCCCAGGTCACGATAGACGCCAG-3′
ExbBdelextss1	5′P- GAAACCCGCGGCATGGACCTGTCCATTTGGGG-3′
ExbBdelextss2	5′P- TGCCTGCGCGCTGCCGGCCAGCCCCACAA-3′

Table S2: list of strains, plasmids and oligonucleotide sequences used in this work
