# Supplementary material for: Structural and molecular determinants for the interaction of ExbB from Serratia marcescens and HasB, a TonB paralog
Source: Commun Biol. 2022 Apr 13;5:355. doi: 10.1038/s42003-022-03306-y (PMC9008036; doi:10.1038/s42003-022-03306-y)
Supplement: Supplementary file 3 — Description of Additional Supplementary Files [file 42003_2022_3306_MOESM3_ESM.pdf]

Description of Additional Supplementary Files

File name: Supplementary Data 1
Description: Source data for Figure 3d.

File name: Supplementary Data 2
Description: Source data for Figure 5a.
