# Supplementary material for: Structural and molecular determinants for the interaction of ExbB from Serratia marcescens and HasB, a TonB paralog
Source: Commun Biol. 2022 Apr 13;5:355. doi: 10.1038/s42003-022-03306-y (PMC9008036; doi:10.1038/s42003-022-03306-y)
Supplement: Supplementary file 5 — Supplementary Data 2 [file 42003_2022_3306_MOESM5_ESM.pdf]

User: USER Path: C:\Prog Test run no.: 335
Test name: C Date: 26/10/ Time: 14:47:17

ID1: isradeb-isradeExbBD
Absorbance
columns X1, X2: strain E. coli C600ΔhemAΔtonBΔexbBD(pAMHasISRADEB+pBAD24)

columns X3, X4: strain E. coli C600ΔhemAΔexbBD(pAMHasISRADE+pBAD24)
columns X19, X37: strain E. coli C600ΔhemAΔtonBΔexbBD(pAMHasISRADEB+pBAD24ExbBDSm)
columns X20, X38: strain E. coli C600ΔhemAΔtonBΔexbBD(pAMHasISRADEB+pBAD24ExbBDEc)
columns X25, X31: strain E. coli C600ΔhemAΔexbBD(pAMHasISRADE+pBAD24ExbBDSm)
columns X26, X32: strain E. coli C600ΔhemAΔexbBD(pAMHasISRADE+pBAD24ExbBDEc)

Well		A01	A02	A03	A04	A05	A06	A07	A08	B01	B02	B03	B04	B05	B06	B07	B08
Content	Time	Blank B	Sample X1	Sample X2	Blank B	Blank B	Blank B	Blank B	Blank B	Blank B	Sample X19	Sample X20	Sample X21	Sample X22	Sample X23	Sample X24	Blank B
Raw Data (6(	0 h	0.204	0.193	0.188	0.194	0.194	0.186	0.188	0.197	0.187	0.172	0.17	0.172	0.172	0.169	0.166	0.172
Raw Data (6(	0 h 30 min	0.182	0.181	0.175	0.177	0.172	0.171	0.167	0.174	0.173	0.166	0.163	0.166	0.166	0.162	0.158	0.165
Raw Data (6(	1 h	0.179	0.179	0.177	0.178	0.173	0.172	0.168	0.174	0.17	0.163	0.162	0.162	0.16	0.158	0.155	0.159
Raw Data (6(	1 h 30 min	0.178	0.177	0.174	0.174	0.174	0.176	0.169	0.178	0.169	0.165	0.163	0.164	0.162	0.158	0.156	0.161
Raw Data (6(	2 h	0.18	0.181	0.178	0.182	0.178	0.179	0.176	0.179	0.167	0.163	0.161	0.164	0.162	0.159	0.157	0.158
Raw Data (6(	2 h 30 min	0.18	0.176	0.175	0.176	0.172	0.171	0.17	0.178	0.166	0.164	0.161	0.163	0.162	0.159	0.157	0.16
Raw Data (6(	3 h	0.179	0.177	0.174	0.174	0.172	0.172	0.17	0.179	0.164	0.166	0.161	0.163	0.162	0.158	0.154	0.156
Raw Data (6(	3 h 30 min	0.178	0.175	0.171	0.171	0.167	0.168	0.166	0.171	0.166	0.169	0.164	0.166	0.166	0.163	0.16	0.163
Raw Data (6(	4 h	0.178	0.177	0.174	0.174	0.17	0.172	0.166	0.176	0.166	0.17	0.164	0.165	0.164	0.16	0.158	0.159
Raw Data (6(	4 h 30 min	0.176	0.177	0.175	0.175	0.172	0.17	0.166	0.178	0.165	0.174	0.165	0.168	0.166	0.162	0.16	0.16
Raw Data (6(	5 h	0.178	0.178	0.177	0.175	0.17	0.167	0.166	0.176	0.165	0.176	0.168	0.169	0.168	0.165	0.163	0.16
Raw Data (6(	5 h 30 min	0.181	0.18	0.178	0.176	0.174	0.174	0.172	0.174	0.165	0.179	0.172	0.172	0.17	0.166	0.162	0.161
Raw Data (6(	6 h	0.178	0.181	0.18	0.175	0.175	0.174	0.172	0.181	0.165	0.184	0.174	0.173	0.173	0.168	0.166	0.16
Raw Data (6(	6 h 30 min	0.18	0.185	0.18	0.175	0.172	0.171	0.167	0.177	0.166	0.188	0.173	0.174	0.175	0.172	0.17	0.159
Raw Data (6(	7 h	0.177	0.183	0.18	0.172	0.169	0.173	0.17	0.182	0.166	0.193	0.177	0.178	0.176	0.173	0.17	0.161
Raw Data (6(	7 h 30 min	0.179	0.189	0.184	0.176	0.172	0.172	0.17	0.177	0.168	0.199	0.175	0.177	0.174	0.169	0.168	0.157
Raw Data (6(	8 h	0.18	0.188	0.186	0.175	0.173	0.174	0.169	0.175	0.165	0.214	0.177	0.18	0.178	0.175	0.173	0.161
Raw Data (6(	8 h 30 min	0.18	0.19	0.188	0.175	0.176	0.172	0.17	0.181	0.166	0.234	0.177	0.182	0.177	0.173	0.171	0.158
Raw Data (6(	9 h	0.18	0.191	0.189	0.175	0.174	0.173	0.17	0.181	0.165	0.257	0.178	0.186	0.178	0.175	0.172	0.16
Raw Data (6(	9 h 30 min	0.183	0.193	0.19	0.177	0.175	0.171	0.169	0.183	0.169	0.276	0.18	0.194	0.181	0.178	0.175	0.163
Raw Data (6(	10 h	0.183	0.195	0.19	0.176	0.172	0.173	0.168	0.177	0.172	0.301	0.18	0.202	0.18	0.179	0.175	0.166
Raw Data (6(	10 h 30 min	0.183	0.194	0.19	0.176	0.172	0.172	0.168	0.183	0.167	0.337	0.178	0.214	0.18	0.177	0.175	0.161
Raw Data (6(	11 h	0.185	0.194	0.191	0.179	0.17	0.172	0.17	0.185	0.168	0.382	0.18	0.235	0.18	0.178	0.173	0.163
Raw Data (6(	11 h 30 min	0.184	0.196	0.192	0.179	0.175	0.174	0.172	0.188	0.17	0.42	0.181	0.254	0.182	0.182	0.18	0.166
Raw Data (6(	12 h	0.186	0.197	0.194	0.18	0.179	0.179	0.178	0.19	0.168	0.468	0.18	0.266	0.18	0.18	0.175	0.159
Raw Data (6(	12 h 30 min	0.187	0.197	0.196	0.178	0.174	0.174	0.176	0.189	0.167	0.508	0.18	0.291	0.18	0.183	0.181	0.163
Raw Data (6(	13 h	0.186	0.194	0.192	0.175	0.172	0.173	0.171	0.186	0.168	0.535	0.18	0.328	0.18	0.188	0.183	0.164
Raw Data (6(	13 h 30 min	0.182	0.195	0.192	0.175	0.17	0.172	0.168	0.184	0.169	0.56	0.18	0.363	0.182	0.194	0.188	0.165
Raw Data (6(	14 h	0.189	0.199	0.198	0.18	0.182	0.183	0.179	0.194	0.168	0.579	0.179	0.395	0.177	0.198	0.19	0.159
Raw Data (6(	14 h 30 min	0.187	0.198	0.195	0.178	0.174	0.173	0.169	0.183	0.168	0.602	0.181	0.436	0.182	0.216	0.201	0.16
Raw Data (6(	15 h	0.187	0.198	0.194	0.176	0.175	0.175	0.173	0.185	0.169	0.621	0.182	0.465	0.181	0.236	0.215	0.163
Raw Data (6(	15 h 30 min	0.186	0.199	0.195	0.179	0.175	0.179	0.176	0.191	0.169	0.641	0.182	0.486	0.182	0.255	0.236	0.164
Raw Data (6(	16 h	0.188	0.198	0.196	0.18	0.172	0.173	0.172	0.19	0.17	0.657	0.184	0.503	0.183	0.274	0.253	0.165
Raw Data (6(	16 h 30 min	0.187	0.197	0.194	0.179	0.176	0.179	0.176	0.195	0.168	0.676	0.187	0.522	0.184	0.306	0.27	0.163
Raw Data (6(	17 h	0.186	0.2	0.196	0.177	0.176	0.179	0.176	0.197	0.166	0.694	0.188	0.537	0.181	0.351	0.298	0.164
Raw Data (6(	17 h 30 min	0.191	0.2	0.194	0.177	0.174	0.173	0.17	0.189	0.169	0.713	0.191	0.553	0.186	0.386	0.343	0.168
Raw Data (6(	18 h	0.189	0.2	0.197	0.183	0.18	0.181	0.18	0.193	0.167	0.726	0.196	0.566	0.185	0.425	0.373	0.161
Raw Data (6(	18 h 30 min	0.194	0.202	0.194	0.178	0.175	0.174	0.169	0.189	0.172	0.744	0.204	0.58	0.188	0.469	0.413	0.162
Raw Data (6(	19 h	0.196	0.204	0.199	0.182	0.176	0.174	0.173	0.191	0.171	0.758	0.216	0.594	0.193	0.507	0.458	0.169
Raw Data (6(	19 h 30 min	0.192	0.202	0.198	0.181	0.176	0.182	0.178	0.204	0.169	0.77	0.232	0.608	0.197	0.523	0.491	0.165
Raw Data (6(	20 h	0.191	0.202	0.197	0.178	0.176	0.179	0.177	0.192	0.172	0.784	0.258	0.623	0.206	0.543	0.516	0.166
Raw Data (6(	20 h 30 min	0.195	0.202	0.196	0.181	0.179	0.179	0.177	0.198	0.17	0.789	0.274	0.632	0.215	0.556	0.528	0.167
Raw Data (6(	21 h	0.202	0.203	0.199	0.181	0.176	0.177	0.177	0.202	0.168	0.797	0.292	0.645	0.23	0.57	0.543	0.168
Raw Data (6(	21 h 30 min	0.195	0.2	0.195	0.179	0.179	0.179	0.177	0.2	0.169	0.805	0.318	0.657	0.25	0.584	0.557	0.162
Raw Data (6(	22 h	0.193	0.201	0.196	0.178	0.175	0.175	0.172	0.191	0.172	0.812	0.33	0.668	0.268	0.601	0.571	0.162
Raw Data (6(	22 h 30 min	0.195	0.201	0.196	0.179	0.171	0.177	0.173	0.199	0.172	0.82	0.341	0.682	0.284	0.614	0.585	0.165
Raw Data (6(	23 h	0.203	0.209	0.203	0.184	0.179	0.183	0.178	0.196	0.174	0.83	0.351	0.694	0.314	0.626	0.599	0.167
Raw Data (6(	23 h 30 min	0.197	0.201	0.198	0.178	0.179	0.18	0.177	0.207	0.172	0.837	0.364	0.702	0.349	0.639	0.614	0.172
Raw Data (6(	24 h	0.194	0.203	0.198	0.179	0.18	0.184	0.184	0.212	0.17	0.842	0.372	0.71	0.37	0.649	0.622	0.164
Raw Data (6(	24 h 30 min	0.193	0.199	0.194	0.173	0.173	0.175	0.181	0.201	0.17	0.845	0.38	0.718	0.39	0.662	0.634	0.166
Raw Data (6(	25 h	0.197	0.205	0.2	0.182	0.18	0.181	0.183	0.206	0.17	0.854	0.39	0.729	0.411	0.674	0.646	0.167
Raw Data (6(	25 h 30 min	0.201	0.204	0.197	0.179	0.178	0.181	0.178	0.206	0.172	0.858	0.398	0.739	0.427	0.684	0.657	0.167
Raw Data (6(	26 h	0.202	0.204	0.197	0.18	0.176	0.177	0.175	0.204	0.173	0.865	0.407	0.742	0.443	0.698	0.669	0.171
Raw Data (6(	26 h 30 min	0.205	0.205	0.196	0.182	0.178	0.181	0.178	0.209	0.173	0.868	0.411	0.753	0.458	0.709	0.677	0.165
Raw Data (6(	27 h	0.199	0.202	0.197	0.181	0.179	0.181	0.179	0.208	0.172	0.874	0.417	0.768	0.468	0.72	0.692	0.169
Raw Data (6(	27 h 30 min	0.196	0.202	0.197	0.18	0.176	0.179	0.178	0.201	0.173	0.88	0.426	0.778	0.474	0.729	0.702	0.171
Raw Data (6(	28 h	0.201	0.203	0.197	0.179	0.182	0.184	0.183	0.208	0.172	0.882	0.427	0.782	0.48	0.739	0.711	0.166
Raw Data (6(	28 h 30 min	0.202	0.206	0.199	0.18	0.179	0.182	0.18	0.207	0.182	0.893	0.439	0.786	0.488	0.75	0.723	0.171
Raw Data (6(	29 h	0.207	0.212	0.205	0.187	0.192	0.188	0.19	0.219	0.174	0.896	0.439	0.792	0.491	0.755	0.728	0.171
Raw Data (6(	29 h 30 min	0.202	0.203	0.198	0.183	0.178	0.183	0.182	0.218	0.173	0.898	0.444	0.807	0.499	0.765	0.738	0.177
Raw Data (6(	30 h	0.208	0.209	0.201	0.185	0.188	0.193	0.19	0.223	0.172	0.902	0.448	0.814	0.502	0.769	0.744	0.168
Raw Data (6(	30 h 30 min	0.2	0.205	0.197	0.178	0.182	0.186	0.183	0.21	0.172	0.904	0.452	0.814	0.508	0.773	0.748	0.168
Raw Data (6(	31 h	0.207	0.207	0.198	0.181	0.182	0.181	0.182	0.213	0.178	0.909	0.458	0.814	0.515	0.784	0.76	0.177
Raw Data (6(	31 h 30 min	0.21	0.207	0.201	0.185	0.184	0.187	0.184	0.215	0.174	0.912	0.462	0.827	0.519	0.792	0.768	0.175
Raw Data (6(	32 h	0.204	0.209	0.202	0.183	0.181	0.185	0.184	0.214	0.173	0.914	0.465	0.834	0.523	0.795	0.77	0.171
Raw Data (6(	32 h 30 min	0.21	0.205	0.197	0.182	0.182	0.187	0.182	0.222	0.177	0.922	0.473	0.84	0.531	0.804	0.777	0.175
Raw Data (6(	33 h	0.213	0.212	0.205	0.196	0.195	0.199	0.201	0.236	0.173	0.92	0.474	0.832	0.532	0.809	0.777	0.173
Raw Data (6(	33 h 30 min	0.215	0.207	0.2	0.183	0.179	0.183	0.182	0.218	0.177	0.925	0.479	0.848	0.538	0.816	0.789	0.178
Raw Data (6(	34 h	0.211	0.205	0.198	0.181	0.181	0.187	0.18	0.222	0.172	0.928	0.479	0.853	0.54	0.821	0.795	0.171
Raw Data (6(	34 h 30 min	0.212	0.204	0.196	0.182	0.185	0.186	0.185	0.227	0.174	0.93	0.483	0.853	0.544	0.824	0.799	0.171
Raw Data (6(	35 h	0.209	0.205	0.2	0.182	0.18	0.184	0.182	0.221	0.176	0.934	0.489	0.85	0.549	0.83	0.808	0.178
Raw Data (6(	35 h 30 min	0.21	0.206	0.201	0.182	0.184	0.188	0.187	0.226	0.176	0.935	0.494	0.855	0.551	0.836	0.817	0.172
Raw Data (6(	36 h	0.216	0.206	0.199	0.186	0.187	0.19	0.192	0.221	0.176	0.938	0.496	0.869	0.559	0.84	0.814	0.175
Raw Data (6(	36 h 30 min	0.216	0.21	0.204	0.187	0.189	0.19	0.196	0.229	0.174	0.938	0.499	0.869	0.56	0.843	0.824	0.174
Raw Data (6(	37 h	0.215	0.211	0.203	0.184	0.187	0.192	0.192	0.226	0.175	0.943	0.505	0.87	0.564	0.839	0.821	0.169
Raw Data (6(	37 h 30 min	0.219	0.208	0.202	0.187	0.185	0.19	0.191	0.228	0.175	0.944	0.509	0.872	0.567	0.845	0.834	0.173
Raw Data (6(	38 h	0.222	0.208	0.2	0.185	0.181	0.187	0.186	0.224	0.178	0.947	0.513	0.879	0.576	0.854	0.839	0.181
Raw Data (6(	38 h 30 min	0.221	0.208	0.2	0.185	0.183	0.189	0.187	0.228	0.177	0.947	0.516	0.884	0.576	0.858	0.843	0.173
Raw Data (6(	39 h	0.22	0.208	0.202	0.183	0.184	0.187	0.188	0.23	0.176	0.946	0.517	0.881	0.576	0.86	0.839	0.166
Raw Data (6(	39 h 30 min	0.217	0.209	0.201	0.185	0.186	0.195	0.193	0.231	0.178	0.951	0.523	0.883	0.582	0.866	0.846	0.175
Raw Data (6(	40 h	0.22	0.211	0.204	0.185	0.188	0.196	0.197	0.238	0.176	0.952	0.526	0.893	0.585	0.87	0.851	0.175
Raw Data (6(	40 h 30 min	0.222	0.21	0.201	0.184	0.182	0.192	0.191	0.236	0.18	0.955	0.53	0.89	0.589	0.875	0.853	0.178
Raw Data (6(	41 h	0.23	0.21	0.202	0.183	0.185	0.192	0.191	0.237	0.18	0.955	0.532	0.891	0.592	0.875	0.854	0.183
Raw Data (6(	41 h 30 min	0.225	0.212	0.203	0.186	0.185	0.188	0.19	0.238	0.178	0.956	0.532	0.894	0.592	0.875	0.853	0.169
Raw Data (6(	42 h	0.226	0.208	0.201	0.186	0.186	0.193	0.194	0.236	0.18	0.958	0.537	0.903	0.599	0.88	0.86	0.177
Raw Data (6(	42 h 30 min	0.224	0.206	0.201	0.183	0.185	0.19	0.19	0.233	0.178	0.957	0.537	0.9	0.599	0.881	0.863	0.174
Raw Data (6(	43 h	0.232	0.211	0.202	0.185	0.183	0.19	0.19	0.236	0.182	0.959	0.544	0.894	0.603	0.883	0.865	0.178
Raw Data (6(	43 h 30 min	0.231	0.214	0.205	0.189	0.189	0.192	0.193	0.238	0.181	0.961	0.547	0.899	0.606	0.888	0.871	0.181
Raw Data (6(	44 h	0.229	0.215	0.209	0.192	0.192	0.196	0.197	0.239	0.183	0.963	0.548	0.908	0.607	0.889	0.871	0.176
Raw Data (6(	44 h 30 min	0.236	0.215	0.205	0.189	0.19	0.195	0.196	0.244	0.184	0.965	0.551	0.911	0.61	0.891	0.875	0.179
Raw Data (6(	45 h	0.233	0.208	0.204	0.187	0.193	0.194	0.201	0.257	0.18	0.967	0.552	0.908	0.614	0.893	0.876	0.181
Raw Data (6(	45 h 30 min	0.236	0.214	0.202	0.186	0.189	0.195	0.19	0.248	0.183	0.966	0.555	0.9	0.617	0.897	0.878	0.181
Raw Data (6(	46 h	0.236	0.214	0.203	0.187	0.19	0.196	0.196	0.249	0.184	0.967	0.56	0.91	0.618	0.897	0.881	0.184
Raw Data (6(	46 h 30 min	0.233	0.214	0.205	0.191	0.192	0.2	0.2	0.242	0.185	0.969	0.562	0.918	0.62	0.899	0.88	0.177
Raw Data (6(	47 h	0.243	0.221	0.209	0.192	0.196	0.203	0.205	0.256	0.182	0.971	0.565	0.919	0.622	0.9	0.883	0.176
Raw Data (6(	47 h 30 min	0.238	0.214	0.203	0.189	0.189	0.196	0.195	0.247	0.187	0.97	0.566	0.918	0.625	0.902	0.889	0.186
Raw Data (6(	48 h	0.242	0.221	0.21	0.199	0.2	0.208	0.209	0.263	0.182	0.971	0.571	0.919	0.626	0.904	0.888	0.18
Raw Data (6(	48 h 30 min	0.244	0.216	0.209	0.193	0.194	0.199	0.201	0.244	0.18	0.972	0.572	0.921	0.626	0.907	0.89	0.184
Raw Data (6(	49 h	0.238	0.216	0.206	0.191	0.193	0.2	0.2	0.255	0.184	0.974	0.574	0.922	0.629	0.908	0.889	0.181
Raw Data (6(	49 h 30 min	0.243	0.221	0.209	0.195	0.193	0.2	0.203	0.251	0.181	0.972	0.574	0.919	0.628	0.907	0.89	0.176
Raw Data (6(	50 h	0.24	0.218	0.206	0.19	0.186	0.192	0.194	0.251	0.186	0.973	0.578	0.923	0.632	0.911	0.895	0.186
Raw Data (6(	50 h 30 min	0.243	0.216	0.203	0.189	0.191	0.198	0.203	0.248	0.182	0.974	0.579	0.918	0.63	0.91	0.896	0.185
Raw Data (6(	51 h	0.24	0.22	0.208	0.194	0.197	0.204	0.21	0.258	0.184	0.972	0.582	0.92	0.632	0.911	0.893	0.185
Raw Data (6(	51 h 30 min	0.246	0.215	0.205	0.19	0.188	0.194	0.196	0.252	0.182	0.972	0.584	0.927	0.633	0.916	0.899	0.181
Raw Data (6(	52 h	0.246	0.22	0.209	0.197	0.197	0.204	0.208	0.256	0.184	0.972	0.584	0.925	0.63	0.912	0.895	0.181
Raw Data (6(	52 h 30 min	0.245	0.223	0.204	0.188	0.192	0.201	0.202	0.255	0.184	0.971	0.586	0.924	0.632	0.914	0.898	0.188
Raw Data (6(	53 h	0.246	0.221	0.205	0.189	0.188	0.2	0.198	0.25	0.186	0.974	0.588	0.923	0.634	0.915	0.901	0.19
Raw Data (6(	53 h 30 min	0.257	0.225	0.208	0.19	0.19	0.2	0.207	0.258	0.184	0.97	0.586	0.922	0.63	0.914	0.898	0.181
Raw Data (6(	54 h	0.247	0.215	0.201	0.187	0.187	0.194	0.198	0.246	0.187	0.97	0.587	0.922	0.631	0.915	0.9	0.18
Raw Data (6(	54 h 30 min	0.247	0.22	0.204	0.19	0.194	0.198	0.202	0.259	0.185	0.968	0.589	0.918	0.631	0.914	0.898	0.182
Raw Data (6(	55 h	0.255	0.222	0.207	0.192	0.193	0.201	0.206	0.266	0.186	0.968	0.589	0.921	0.631	0.917	0.901	0.183
Raw Data (6(	55 h 30 min	0.25	0.223	0.209	0.192	0.195	0.2	0.205	0.256	0.186	0.971	0.592	0.923	0.631	0.916	0.899	0.179
Raw Data (6(	56 h	0.25	0.222	0.21	0.193	0.196	0.205	0.211	0.262	0.189	0.973	0.593	0.925	0.633	0.92	0.906	0.183
Raw Data (6(	56 h 30 min	0.248	0.219	0.205	0.191	0.19	0.201	0.202	0.26	0.185	0.974	0.596	0.924	0.634	0.922	0.908	0.191
Raw Data (6(	57 h	0.252	0.221	0.204	0.194	0.197	0.206	0.21	0.274	0.188	0.974	0.593	0.923	0.632	0.917	0.902	0.185
Raw Data (6(	57 h 30 min	0.251	0.224	0.207	0.194	0.198	0.204	0.204	0.272	0.188	0.973	0.596	0.928	0.633	0.919	0.906	0.188
Raw Data (6(	58 h	0.247	0.218	0.204	0.189	0.194	0.209	0.209	0.264	0.19	0.969	0.594	0.921	0.633	0.921	0.905	0.185
Raw Data (6(	58 h 30 min	0.251	0.224	0.208	0.194	0.195	0.205	0.207	0.262	0.192	0.972	0.596	0.92	0.633	0.921	0.906	0.184
Raw Data (6(	59 h	0.26	0.227	0.211	0.197	0.198	0.205	0.21	0.269	0.193	0.97	0.594	0.922	0.635	0.921	0.907	0.181
Raw Data (6(	59 h 30 min	0.255	0.224	0.206	0.194	0.196	0.204	0.205	0.26	0.192	0.973	0.6	0.924	0.634	0.923	0.908	0.192
Raw Data (6(	60 h	0.255	0.223	0.208	0.197	0.195	0.204	0.207	0.267	0.192	0.974	0.603	0.924	0.637	0.925	0.912	0.191
Raw Data (6(	60 h 30 min	0.252	0.224	0.214	0.198	0.201	0.211	0.213	0.265	0.191	0.97	0.598	0.921	0.636	0.924	0.912	0.195
Raw Data (6(	61 h	0.263	0.226	0.211	0.198	0.201	0.207	0.214	0.275	0.188	0.971	0.599	0.923	0.636	0.926	0.911	0.191
Raw Data (6(	61 h 30 min	0.258	0.223	0.208	0.198	0.197	0.204	0.212	0.272	0.191	0.971	0.601	0.922	0.637	0.928	0.914	0.193
Raw Data (6(	62 h	0.264	0.226	0.214	0.197	0.199	0.211	0.216	0.268	0.192	0.966	0.599	0.919	0.634	0.922	0.908	0.179
Raw Data (6(	62 h 30 min	0.26	0.225	0.206	0.194	0.198	0.21	0.211	0.269	0.2	0.967	0.599	0.922	0.634	0.925	0.912	0.191
Raw Data (6(	63 h	0.26	0.227	0.215	0.195	0.199	0.211	0.213	0.269	0.192	0.967	0.6	0.914	0.635	0.924	0.912	0.192
Raw Data (6(	63 h 30 min	0.261	0.231	0.214	0.2	0.2	0.213	0.217	0.273	0.196	0.969	0.603	0.919	0.638	0.928	0.915	0.199
Raw Data (6(	64 h	0.264	0.228	0.211	0.197	0.196	0.207	0.211	0.271	0.191	0.966	0.6	0.911	0.633	0.926	0.913	0.185
Raw Data (6(	64 h 30 min	0.261	0.226	0.21	0.196	0.199	0.208	0.213	0.271	0.192	0.965	0.601	0.91	0.634	0.924	0.912	0.192
Raw Data (6(	65 h	0.261	0.229	0.21	0.197	0.198	0.211	0.214	0.276	0.191	0.966	0.602	0.917	0.635	0.926	0.914	0.196
Raw Data (6(	65 h 30 min	0.264	0.229	0.216	0.202	0.203	0.21	0.217	0.273	0.196	0.966	0.601	0.915	0.636	0.928	0.914	0.195
Raw Data (6(	66 h	0.265	0.231	0.21	0.199	0.203	0.215	0.22	0.281	0.191	0.966	0.604	0.918	0.635	0.932	0.916	0.202
Raw Data (6(	66 h 30 min	0.258	0.228	0.21	0.196	0.2	0.217	0.214	0.27	0.201	0.964	0.603	0.912	0.635	0.928	0.913	0.196

Well		C01	C02	C03	C04	C05	C06	C07	C08	D01	D02	D03	D04	D05	D06	D07	D08
Content	Time	Blank B	Sample X25	Sample X26	Sample X27	Sample X28	Sample X29	Sample X30	Blank B	Blank B	Sample X31	Sample X32	Sample X33	Sample X34	Sample X35	Sample X36	Blank B
Raw Data (6(	0 h	0.18	0.178	0.176	0.178	0.185	0.183	0.181	0.175	0.188	0.175	0.171	0.172	0.171	0.175	0.166	0.172
Raw Data (6(	0 h 30 min	0.169	0.167	0.168	0.167	0.179	0.173	0.167	0.159	0.173	0.169	0.166	0.166	0.164	0.168	0.167	0.167
Raw Data (6(	1 h	0.165	0.166	0.163	0.164	0.173	0.17	0.167	0.16	0.169	0.167	0.164	0.164	0.162	0.187	0.16	0.16
Raw Data (6(	1 h 30 min	0.167	0.165	0.165	0.168	0.178	0.172	0.169	0.16	0.172	0.191	0.197	0.163	0.162	0.164	0.188	0.166
Raw Data (6(	2 h	0.169	0.17	0.179	0.199	0.177	0.169	0.167	0.158	0.17	0.168	0.168	0.18	0.222	0.254	0.206	0.164
Raw Data (6(	2 h 30 min	0.167	0.167	0.166	0.167	0.178	0.173	0.17	0.161	0.169	0.166	0.217	0.228	0.244	0.176	0.167	0.163
Raw Data (6(	3 h	0.167	0.168	0.168	0.167	0.177	0.172	0.172	0.159	0.171	0.167	0.167	0.165	0.165	0.17	0.165	0.168
Raw Data (6(	3 h 30 min	0.165	0.169	0.179	0.173	0.18	0.169	0.17	0.16	0.174	0.195	0.217	0.193	0.164	0.167	0.164	0.168
Raw Data (6(	4 h	0.166	0.171	0.172	0.172	0.186	0.17	0.174	0.161	0.172	0.171	0.17	0.167	0.167	0.168	0.166	0.165
Raw Data (6(	4 h 30 min	0.167	0.171	0.172	0.172	0.187	0.171	0.177	0.164	0.17	0.171	0.174	0.171	0.168	0.169	0.168	0.165
Raw Data (6(	5 h	0.165	0.176	0.176	0.174	0.189	0.168	0.174	0.158	0.171	0.174	0.176	0.172	0.173	0.179	0.176	0.172
Raw Data (6(	5 h 30 min	0.163	0.176	0.178	0.176	0.19	0.17	0.183	0.16	0.166	0.175	0.178	0.174	0.172	0.173	0.175	0.162
Raw Data (6(	6 h	0.168	0.181	0.182	0.178	0.195	0.17	0.183	0.163	0.17	0.179	0.179	0.175	0.174	0.173	0.173	0.163
Raw Data (6(	6 h 30 min	0.167	0.182	0.184	0.18	0.193	0.167	0.181	0.158	0.168	0.183	0.211	0.186	0.191	0.197	0.174	0.163
Raw Data (6(	7 h	0.168	0.182	0.192	0.183	0.196	0.171	0.187	0.163	0.177	0.183	0.202	0.185	0.177	0.178	0.178	0.167
Raw Data (6(	7 h 30 min	0.172	0.188	0.187	0.184	0.198	0.169	0.184	0.16	0.168	0.18	0.183	0.181	0.178	0.178	0.179	0.162
Raw Data (6(	8 h	0.167	0.185	0.185	0.185	0.198	0.172	0.188	0.161	0.17	0.181	0.206	0.217	0.179	0.179	0.178	0.166
Raw Data (6(	8 h 30 min	0.171	0.186	0.186	0.186	0.2	0.171	0.189	0.16	0.17	0.182	0.184	0.181	0.179	0.176	0.175	0.164
Raw Data (6(	9 h	0.172	0.183	0.186	0.186	0.198	0.168	0.185	0.159	0.171	0.184	0.185	0.183	0.183	0.179	0.179	0.166
Raw Data (6(	9 h 30 min	0.165	0.184	0.188	0.208	0.2	0.171	0.188	0.162	0.171	0.184	0.186	0.195	0.18	0.182	0.184	0.169
Raw Data (6(	10 h	0.166	0.187	0.185	0.185	0.199	0.168	0.186	0.16	0.169	0.182	0.184	0.183	0.181	0.182	0.181	0.168
Raw Data (6(	10 h 30 min	0.168	0.187	0.188	0.201	0.216	0.17	0.191	0.162	0.17	0.185	0.186	0.2	0.234	0.181	0.182	0.168
Raw Data (6(	11 h	0.169	0.19	0.186	0.186	0.198	0.166	0.185	0.158	0.171	0.185	0.187	0.187	0.183	0.184	0.179	0.167
Raw Data (6(	11 h 30 min	0.167	0.186	0.188	0.188	0.2	0.169	0.191	0.162	0.169	0.186	0.187	0.186	0.182	0.184	0.181	0.173
Raw Data (6(	12 h	0.168	0.188	0.189	0.188	0.2	0.169	0.19	0.16	0.17	0.186	0.187	0.185	0.188	0.22	0.186	0.166
Raw Data (6(	12 h 30 min	0.165	0.187	0.189	0.187	0.223	0.198	0.197	0.164	0.173	0.187	0.188	0.185	0.213	0.222	0.189	0.166
Raw Data (6(	13 h	0.166	0.189	0.189	0.191	0.201	0.169	0.19	0.161	0.171	0.186	0.188	0.22	0.248	0.227	0.185	0.166
Raw Data (6(	13 h 30 min	0.166	0.188	0.19	0.19	0.202	0.173	0.193	0.162	0.176	0.191	0.192	0.264	0.196	0.188	0.186	0.169
Raw Data (6(	14 h	0.167	0.187	0.189	0.187	0.201	0.17	0.192	0.163	0.172	0.188	0.195	0.19	0.184	0.182	0.18	0.162
Raw Data (6(	14 h 30 min	0.171	0.193	0.197	0.198	0.204	0.172	0.195	0.163	0.173	0.19	0.208	0.201	0.186	0.185	0.182	0.167
Raw Data (6(	15 h	0.174	0.203	0.225	0.192	0.203	0.168	0.192	0.16	0.169	0.2	0.255	0.223	0.211	0.182	0.188	0.166
Raw Data (6(	15 h 30 min	0.167	0.188	0.195	0.19	0.206	0.171	0.199	0.162	0.171	0.189	0.194	0.225	0.221	0.185	0.189	0.17
Raw Data (6(	16 h	0.173	0.193	0.2	0.191	0.207	0.17	0.199	0.162	0.172	0.189	0.195	0.189	0.19	0.186	0.189	0.168
Raw Data (6(	16 h 30 min	0.168	0.19	0.204	0.19	0.209	0.171	0.203	0.164	0.172	0.19	0.201	0.189	0.192	0.185	0.188	0.166
Raw Data (6(	17 h	0.166	0.187	0.208	0.189	0.214	0.172	0.208	0.164	0.17	0.189	0.206	0.189	0.196	0.187	0.193	0.172
Raw Data (6(	17 h 30 min	0.166	0.19	0.218	0.189	0.22	0.171	0.212	0.162	0.174	0.192	0.215	0.19	0.213	0.189	0.201	0.171
Raw Data (6(	18 h	0.168	0.191	0.235	0.193	0.228	0.173	0.228	0.167	0.176	0.191	0.229	0.193	0.223	0.191	0.208	0.171
Raw Data (6(	18 h 30 min	0.17	0.19	0.257	0.19	0.243	0.171	0.24	0.165	0.174	0.19	0.247	0.191	0.23	0.19	0.22	0.174
Raw Data (6(	19 h	0.17	0.194	0.285	0.192	0.261	0.169	0.258	0.162	0.17	0.188	0.272	0.188	0.247	0.19	0.23	0.17
Raw Data (6(	19 h 30 min	0.169	0.192	0.302	0.192	0.288	0.171	0.281	0.164	0.171	0.188	0.294	0.188	0.271	0.188	0.245	0.167
Raw Data (6(	20 h	0.172	0.195	0.327	0.191	0.309	0.169	0.3	0.162	0.173	0.19	0.319	0.226	0.294	0.191	0.269	0.176
Raw Data (6(	20 h 30 min	0.172	0.194	0.341	0.191	0.331	0.168	0.317	0.161	0.172	0.192	0.335	0.305	0.357	0.189	0.287	0.174
Raw Data (6(	21 h	0.171	0.194	0.355	0.194	0.364	0.194	0.328	0.164	0.175	0.193	0.343	0.204	0.432	0.201	0.301	0.175
Raw Data (6(	21 h 30 min	0.175	0.195	0.372	0.194	0.361	0.171	0.341	0.163	0.173	0.19	0.358	0.189	0.346	0.195	0.308	0.168
Raw Data (6(	22 h	0.168	0.2	0.412	0.194	0.376	0.17	0.353	0.162	0.177	0.194	0.389	0.194	0.353	0.194	0.322	0.178
Raw Data (6(	22 h 30 min	0.168	0.199	0.422	0.193	0.403	0.171	0.372	0.166	0.172	0.195	0.407	0.19	0.372	0.196	0.334	0.175
Raw Data (6(	23 h	0.172	0.202	0.451	0.196	0.436	0.189	0.397	0.163	0.176	0.198	0.452	0.201	0.416	0.279	0.411	0.175
Raw Data (6(	23 h 30 min	0.174	0.206	0.488	0.195	0.472	0.17	0.426	0.164	0.175	0.198	0.481	0.228	0.421	0.286	0.378	0.176
Raw Data (6(	24 h	0.17	0.211	0.511	0.197	0.512	0.17	0.463	0.166	0.173	0.205	0.515	0.192	0.451	0.216	0.435	0.177
Raw Data (6(	24 h 30 min	0.178	0.22	0.524	0.199	0.533	0.171	0.501	0.163	0.173	0.205	0.524	0.193	0.485	0.202	0.438	0.176
Raw Data (6(	25 h	0.173	0.225	0.536	0.202	0.547	0.172	0.528	0.166	0.177	0.212	0.536	0.216	0.551	0.21	0.478	0.178
Raw Data (6(	25 h 30 min	0.174	0.239	0.545	0.204	0.559	0.171	0.54	0.164	0.173	0.22	0.548	0.197	0.521	0.265	0.52	0.171
Raw Data (6(	26 h	0.17	0.256	0.556	0.206	0.57	0.168	0.553	0.165	0.174	0.234	0.592	0.244	0.526	0.229	0.516	0.175
Raw Data (6(	26 h 30 min	0.178	0.28	0.577	0.24	0.59	0.17	0.567	0.164	0.172	0.25	0.636	0.264	0.535	0.241	0.529	0.165
Raw Data (6(	27 h	0.17	0.292	0.579	0.218	0.596	0.173	0.583	0.167	0.172	0.274	0.583	0.202	0.547	0.263	0.547	0.175
Raw Data (6(	27 h 30 min	0.173	0.298	0.589	0.224	0.608	0.17	0.594	0.164	0.175	0.295	0.594	0.203	0.556	0.286	0.558	0.174
Raw Data (6(	28 h	0.171	0.304	0.599	0.231	0.62	0.17	0.608	0.164	0.174	0.304	0.605	0.233	0.569	0.305	0.573	0.175
Raw Data (6(	28 h 30 min	0.175	0.317	0.609	0.243	0.632	0.172	0.62	0.165	0.176	0.314	0.617	0.215	0.579	0.308	0.585	0.176
Raw Data (6(	29 h	0.173	0.325	0.62	0.256	0.644	0.17	0.632	0.164	0.177	0.323	0.628	0.218	0.589	0.32	0.599	0.178
Raw Data (6(	29 h 30 min	0.17	0.338	0.629	0.267	0.655	0.169	0.643	0.162	0.174	0.346	0.644	0.221	0.604	0.377	0.663	0.178
Raw Data (6(	30 h	0.172	0.356	0.637	0.276	0.67	0.173	0.656	0.171	0.174	0.35	0.67	0.231	0.608	0.333	0.622	0.175
Raw Data (6(	30 h 30 min	0.167	0.374	0.647	0.279	0.68	0.172	0.669	0.167	0.182	0.372	0.66	0.244	0.621	0.353	0.637	0.183
Raw Data (6(	31 h	0.17	0.395	0.659	0.281	0.691	0.169	0.68	0.166	0.176	0.391	0.744	0.3	0.684	0.37	0.657	0.175
Raw Data (6(	31 h 30 min	0.174	0.415	0.666	0.286	0.701	0.17	0.691	0.165	0.178	0.411	0.772	0.38	0.744	0.433	0.661	0.178
Raw Data (6(	32 h	0.168	0.434	0.678	0.286	0.711	0.169	0.701	0.168	0.181	0.432	0.69	0.276	0.652	0.407	0.672	0.178
Raw Data (6(	32 h 30 min	0.175	0.454	0.687	0.292	0.718	0.173	0.707	0.168	0.177	0.45	0.697	0.282	0.659	0.427	0.678	0.175
Raw Data (6(	33 h	0.175	0.46	0.693	0.299	0.724	0.169	0.712	0.166	0.171	0.456	0.704	0.285	0.692	0.476	0.691	0.178
Raw Data (6(	33 h 30 min	0.175	0.466	0.698	0.309	0.727	0.17	0.721	0.166	0.177	0.463	0.708	0.282	0.675	0.452	0.69	0.183
Raw Data (6(	34 h	0.177	0.474	0.701	0.321	0.733	0.172	0.727	0.169	0.179	0.47	0.714	0.303	0.749	0.498	0.702	0.184
Raw Data (6(	34 h 30 min	0.176	0.481	0.707	0.335	0.735	0.17	0.73	0.168	0.176	0.478	0.719	0.291	0.695	0.466	0.706	0.181
Raw Data (6(	35 h	0.17	0.486	0.71	0.348	0.75	0.197	0.734	0.169	0.173	0.484	0.723	0.294	0.687	0.491	0.711	0.183
Raw Data (6(	35 h 30 min	0.177	0.492	0.715	0.363	0.744	0.172	0.737	0.167	0.174	0.509	0.728	0.303	0.696	0.481	0.716	0.181
Raw Data (6(	36 h	0.173	0.498	0.718	0.394	0.769	0.17	0.741	0.167	0.182	0.498	0.735	0.374	0.767	0.486	0.719	0.181
Raw Data (6(	36 h 30 min	0.176	0.504	0.735	0.407	0.756	0.17	0.746	0.165	0.18	0.506	0.839	0.399	0.811	0.495	0.729	0.181
Raw Data (6(	37 h	0.178	0.507	0.726	0.406	0.753	0.171	0.75	0.163	0.178	0.543	0.768	0.335	0.705	0.494	0.726	0.175
Raw Data (6(	37 h 30 min	0.174	0.512	0.729	0.415	0.762	0.172	0.756	0.17	0.178	0.515	0.746	0.375	0.719	0.504	0.731	0.182
Raw Data (6(	38 h	0.175	0.519	0.732	0.419	0.764	0.171	0.758	0.167	0.178	0.524	0.75	0.362	0.714	0.513	0.739	0.186
Raw Data (6(	38 h 30 min	0.177	0.521	0.738	0.422	0.777	0.187	0.761	0.164	0.179	0.524	0.841	0.462	0.884	0.716	0.777	0.183
Raw Data (6(	39 h	0.175	0.53	0.74	0.424	0.77	0.173	0.766	0.17	0.179	0.532	0.758	0.4	0.76	0.543	0.746	0.181
Raw Data (6(	39 h 30 min	0.179	0.537	0.744	0.428	0.775	0.172	0.768	0.171	0.182	0.54	0.766	0.393	0.723	0.525	0.751	0.187
Raw Data (6(	40 h	0.173	0.541	0.745	0.43	0.786	0.202	0.79	0.171	0.185	0.546	0.764	0.42	0.856	0.589	0.835	0.185
Raw Data (6(	40 h 30 min	0.184	0.543	0.752	0.436	0.78	0.17	0.774	0.168	0.178	0.564	0.766	0.399	0.786	0.537	0.756	0.183
Raw Data (6(	41 h	0.18	0.545	0.753	0.437	0.783	0.171	0.777	0.17	0.178	0.552	0.829	0.457	0.732	0.572	0.758	0.184
Raw Data (6(	41 h 30 min	0.181	0.554	0.758	0.441	0.79	0.172	0.783	0.174	0.177	0.59	0.805	0.402	0.738	0.545	0.762	0.178
Raw Data (6(	42 h	0.178	0.554	0.76	0.442	0.791	0.169	0.784	0.168	0.179	0.562	0.776	0.402	0.74	0.548	0.766	0.182
Raw Data (6(	42 h 30 min	0.177	0.554	0.762	0.444	0.792	0.171	0.785	0.17	0.178	0.628	0.892	0.406	0.741	0.554	0.772	0.181
Raw Data (6(	43 h	0.178	0.557	0.765	0.447	0.803	0.199	0.789	0.17	0.186	0.567	0.782	0.404	0.818	0.616	0.773	0.19
Raw Data (6(	43 h 30 min	0.174	0.556	0.766	0.45	0.816	0.2	0.792	0.17	0.177	0.615	0.785	0.429	0.813	0.633	0.776	0.185
Raw Data (6(	44 h	0.173	0.555	0.769	0.447	0.8	0.171	0.796	0.172	0.178	0.573	0.788	0.404	0.749	0.562	0.777	0.186
Raw Data (6(	44 h 30 min	0.183	0.559	0.774	0.452	0.802	0.17	0.796	0.17	0.187	0.574	0.792	0.404	0.758	0.589	0.783	0.19
Raw Data (6(	45 h	0.177	0.558	0.774	0.451	0.804	0.171	0.801	0.173	0.176	0.576	0.891	0.504	0.753	0.563	0.779	0.186
Raw Data (6(	45 h 30 min	0.183	0.564	0.777	0.454	0.807	0.171	0.802	0.173	0.182	0.59	0.87	0.426	0.758	0.568	0.785	0.187
Raw Data (6(	46 h	0.181	0.563	0.779	0.454	0.81	0.174	0.807	0.175	0.182	0.578	0.798	0.418	0.765	0.571	0.788	0.188
Raw Data (6(	46 h 30 min	0.174	0.564	0.779	0.453	0.81	0.173	0.806	0.175	0.188	0.58	0.799	0.404	0.766	0.574	0.791	0.198
Raw Data (6(	47 h	0.179	0.567	0.784	0.454	0.815	0.168	0.808	0.167	0.182	0.583	0.803	0.434	0.808	0.573	0.793	0.186
Raw Data (6(	47 h 30 min	0.187	0.569	0.786	0.457	0.816	0.17	0.812	0.172	0.181	0.583	0.803	0.4	0.775	0.573	0.793	0.184
Raw Data (6(	48 h	0.174	0.57	0.802	0.457	0.817	0.172	0.814	0.175	0.181	0.588	0.844	0.438	0.771	0.578	0.795	0.187
Raw Data (6(	48 h 30 min	0.182	0.569	0.787	0.459	0.822	0.172	0.815	0.173	0.181	0.59	0.81	0.401	0.772	0.58	0.798	0.19
Raw Data (6(	49 h	0.18	0.569	0.789	0.459	0.822	0.175	0.818	0.174	0.184	0.588	0.809	0.4	0.774	0.585	0.797	0.187
Raw Data (6(	49 h 30 min	0.183	0.572	0.791	0.46	0.823	0.168	0.819	0.17	0.178	0.59	0.811	0.4	0.776	0.584	0.802	0.182
Raw Data (6(	50 h	0.179	0.573	0.789	0.46	0.824	0.17	0.821	0.172	0.183	0.654	0.815	0.398	0.777	0.58	0.802	0.189
Raw Data (6(	50 h 30 min	0.175	0.572	0.793	0.461	0.826	0.171	0.826	0.174	0.196	0.593	0.814	0.401	0.779	0.584	0.804	0.197
Raw Data (6(	51 h	0.181	0.572	0.794	0.46	0.826	0.173	0.824	0.17	0.186	0.594	0.818	0.398	0.781	0.585	0.806	0.188
Raw Data (6(	51 h 30 min	0.184	0.575	0.796	0.462	0.826	0.17	0.825	0.174	0.184	0.596	0.818	0.396	0.78	0.586	0.805	0.191
Raw Data (6(	52 h	0.178	0.576	0.798	0.462	0.83	0.17	0.828	0.176	0.186	0.596	0.818	0.396	0.783	0.585	0.809	0.188
Raw Data (6(	52 h 30 min	0.187	0.577	0.795	0.462	0.827	0.168	0.825	0.172	0.188	0.598	0.819	0.395	0.783	0.589	0.81	0.189
Raw Data (6(	53 h	0.183	0.58	0.798	0.465	0.831	0.171	0.83	0.175	0.186	0.599	0.82	0.394	0.784	0.587	0.808	0.19
Raw Data (6(	53 h 30 min	0.181	0.576	0.798	0.463	0.83	0.17	0.827	0.169	0.181	0.6	0.82	0.394	0.785	0.588	0.81	0.182
Raw Data (6(	54 h	0.186	0.578	0.802	0.464	0.832	0.166	0.829	0.173	0.184	0.603	0.822	0.394	0.787	0.59	0.813	0.19
Raw Data (6(	54 h 30 min	0.19	0.581	0.803	0.467	0.833	0.17	0.832	0.179	0.189	0.602	0.823	0.394	0.787	0.593	0.814	0.195
Raw Data (6(	55 h	0.187	0.582	0.802	0.47	0.837	0.171	0.832	0.176	0.185	0.602	0.823	0.395	0.788	0.595	0.811	0.189
Raw Data (6(	55 h 30 min	0.184	0.582	0.802	0.468	0.836	0.174	0.835	0.179	0.187	0.603	0.824	0.395	0.789	0.592	0.813	0.19
Raw Data (6(	56 h	0.185	0.582	0.805	0.472	0.836	0.169	0.834	0.171	0.185	0.605	0.828	0.4	0.792	0.597	0.815	0.186
Raw Data (6(	56 h 30 min	0.187	0.583	0.804	0.472	0.837	0.171	0.836	0.181	0.186	0.605	0.826	0.394	0.79	0.592	0.813	0.186
Raw Data (6(	57 h	0.188	0.582	0.806	0.471	0.837	0.171	0.836	0.173	0.185	0.607	0.828	0.404	0.792	0.594	0.814	0.192
Raw Data (6(	57 h 30 min	0.192	0.584	0.806	0.471	0.838	0.17	0.836	0.176	0.186	0.608	0.826	0.396	0.794	0.597	0.817	0.197
Raw Data (6(	58 h	0.188	0.584	0.807	0.471	0.838	0.165	0.837	0.174	0.19	0.608	0.829	0.394	0.794	0.597	0.819	0.196
Raw Data (6(	58 h 30 min	0.19	0.584	0.809	0.473	0.842	0.176	0.842	0.178	0.192	0.61	0.831	0.394	0.795	0.596	0.819	0.194
Raw Data (6(	59 h	0.19	0.587	0.809	0.471	0.84	0.166	0.839	0.173	0.185	0.613	0.831	0.395	0.797	0.602	0.821	0.188
Raw Data (6(	59 h 30 min	0.188	0.587	0.811	0.476	0.844	0.173	0.844	0.184	0.191	0.611	0.832	0.396	0.799	0.604	0.822	0.196
Raw Data (6(	60 h	0.189	0.588	0.814	0.475	0.845	0.169	0.843	0.176	0.189	0.612	0.832	0.399	0.8	0.603	0.821	0.193
Raw Data (6(	60 h 30 min	0.19	0.589	0.814	0.476	0.845	0.17	0.845	0.176	0.187	0.614	0.836	0.398	0.798	0.6	0.82	0.191
Raw Data (6(	61 h	0.192	0.589	0.813	0.477	0.847	0.171	0.845	0.182	0.188	0.613	0.834	0.399	0.801	0.603	0.822	0.192
Raw Data (6(	61 h 30 min	0.186	0.589	0.813	0.476	0.845	0.172	0.845	0.183	0.189	0.616	0.836	0.4	0.801	0.606	0.824	0.19
Raw Data (6(	62 h	0.196	0.594	0.82	0.479	0.85	0.171	0.847	0.18	0.185	0.615	0.837	0.401	0.804	0.607	0.826	0.184
Raw Data (6(	62 h 30 min	0.191	0.59	0.817	0.477	0.848	0.172	0.847	0.184	0.19	0.618	0.838	0.4	0.802	0.606	0.825	0.2
Raw Data (6(	63 h	0.193	0.592	0.82	0.478	0.851	0.168	0.848	0.178	0.191	0.617	0.839	0.399	0.804	0.607	0.826	0.196
Raw Data (6(	63 h 30 min	0.192	0.594	0.819	0.481	0.853	0.175	0.851	0.193	0.19	0.621	0.839	0.401	0.807	0.609	0.828	0.193
Raw Data (6(	64 h	0.19	0.591	0.818	0.477	0.849	0.164	0.846	0.172	0.189	0.616	0.838	0.397	0.804	0.61	0.826	0.2
Raw Data (6(	64 h 30 min	0.194	0.596	0.819	0.479	0.853	0.17	0.851	0.183	0.194	0.62	0.842	0.434	0.822	0.608	0.827	0.19
Raw Data (6(	65 h	0.192	0.594	0.822	0.48	0.854	0.169	0.852	0.186	0.193	0.621	0.842	0.402	0.809	0.611	0.83	0.199
Raw Data (6(	65 h 30 min	0.194	0.596	0.826	0.48	0.854	0.167	0.853	0.181	0.194	0.624	0.844	0.403	0.809	0.613	0.83	0.205
Raw Data (6(	66 h	0.199	0.598	0.824	0.481	0.855	0.171	0.855	0.188	0.188	0.624	0.844	0.403	0.81	0.615	0.83	0.191
Raw Data (6(	66 h 30 min	0.197	0.602	0.826	0.482	0.857	0.171	0.854	0.183	0.194	0.623	0.863	0.404	0.812	0.615	0.829	0.194

Well		E01	E02	E03	E04	E05	E06	E07	E08	F01	F02	F03	F04	F05	F06	F07	F08
Content	Time	Blank B	Sample X37	Sample X38	Sample X39	Sample X40	Sample X41	Sample X42	Blank B	Blank B	Sample X4	Sample X3	Blank B	Blank B	Blank B	Blank B	Blank B
Raw Data (6(	0 h	0.187	0.177	0.175	0.172	0.17	0.166	0.164	0.163	0.187	0.167	0.165	0.175	0.171	0.161	0.161	0.16
Raw Data (6(	0 h 30 min	0.171	0.166	0.168	0.168	0.165	0.159	0.157	0.158	0.178	0.172	0.165	0.165	0.164	0.155	0.154	0.154
Raw Data (6(	1 h	0.178	0.204	0.171	0.168	0.166	0.171	0.157	0.156	0.168	0.16	0.16	0.161	0.159	0.154	0.152	0.155
Raw Data (6(	1 h 30 min	0.174	0.2	0.255	0.166	0.16	0.151	0.154	0.152	0.179	0.165	0.16	0.159	0.158	0.15	0.149	0.15
Raw Data (6(	2 h	0.17	0.164	0.168	0.166	0.164	0.156	0.155	0.155	0.167	0.161	0.161	0.16	0.159	0.156	0.153	0.154
Raw Data (6(	2 h 30 min	0.168	0.168	0.166	0.165	0.184	0.184	0.154	0.153	0.17	0.163	0.163	0.164	0.162	0.157	0.155	0.155
Raw Data (6(	3 h	0.168	0.168	0.169	0.166	0.166	0.16	0.158	0.159	0.17	0.163	0.161	0.159	0.157	0.152	0.15	0.153
Raw Data (6(	3 h 30 min	0.176	0.17	0.17	0.168	0.163	0.156	0.155	0.153	0.174	0.163	0.162	0.158	0.156	0.15	0.148	0.15
Raw Data (6(	4 h	0.173	0.173	0.171	0.169	0.165	0.157	0.156	0.152	0.172	0.168	0.167	0.165	0.166	0.16	0.156	0.159
Raw Data (6(	4 h 30 min	0.17	0.174	0.174	0.172	0.166	0.16	0.157	0.154	0.169	0.165	0.164	0.159	0.159	0.153	0.151	0.154
Raw Data (6(	5 h	0.172	0.179	0.176	0.172	0.168	0.16	0.16	0.154	0.17	0.166	0.164	0.16	0.159	0.152	0.152	0.152
Raw Data (6(	5 h 30 min	0.174	0.185	0.193	0.178	0.171	0.162	0.16	0.153	0.172	0.168	0.167	0.16	0.159	0.157	0.155	0.156
Raw Data (6(	6 h	0.175	0.184	0.18	0.178	0.173	0.163	0.166	0.153	0.176	0.171	0.168	0.164	0.158	0.156	0.152	0.158
Raw Data (6(	6 h 30 min	0.173	0.189	0.183	0.181	0.177	0.169	0.169	0.158	0.166	0.174	0.174	0.161	0.159	0.153	0.154	0.153
Raw Data (6(	7 h	0.172	0.192	0.182	0.179	0.176	0.168	0.169	0.153	0.181	0.177	0.179	0.162	0.163	0.153	0.154	0.156
Raw Data (6(	7 h 30 min	0.178	0.204	0.187	0.184	0.18	0.17	0.167	0.154	0.168	0.178	0.177	0.16	0.159	0.155	0.152	0.158
Raw Data (6(	8 h	0.176	0.214	0.186	0.182	0.176	0.168	0.168	0.154	0.179	0.181	0.177	0.162	0.161	0.153	0.152	0.156
Raw Data (6(	8 h 30 min	0.172	0.231	0.246	0.265	0.185	0.174	0.172	0.16	0.168	0.177	0.177	0.16	0.159	0.155	0.152	0.157
Raw Data (6(	9 h	0.178	0.257	0.19	0.192	0.183	0.175	0.176	0.164	0.169	0.176	0.18	0.164	0.16	0.157	0.154	0.159
Raw Data (6(	9 h 30 min	0.171	0.272	0.187	0.191	0.178	0.177	0.172	0.153	0.184	0.186	0.184	0.164	0.161	0.154	0.152	0.153
Raw Data (6(	10 h	0.17	0.294	0.186	0.199	0.179	0.171	0.173	0.154	0.172	0.18	0.18	0.161	0.157	0.153	0.15	0.152
Raw Data (6(	10 h 30 min	0.169	0.324	0.186	0.212	0.192	0.176	0.175	0.152	0.168	0.182	0.18	0.16	0.16	0.154	0.152	0.156
Raw Data (6(	11 h	0.175	0.411	0.213	0.231	0.182	0.176	0.176	0.158	0.171	0.182	0.181	0.16	0.16	0.155	0.151	0.156
Raw Data (6(	11 h 30 min	0.174	0.397	0.188	0.248	0.185	0.177	0.178	0.16	0.182	0.186	0.187	0.164	0.161	0.157	0.154	0.154
Raw Data (6(	12 h	0.179	0.438	0.19	0.268	0.199	0.177	0.174	0.153	0.173	0.183	0.182	0.16	0.161	0.156	0.155	0.16
Raw Data (6(	12 h 30 min	0.171	0.482	0.187	0.279	0.182	0.179	0.179	0.158	0.186	0.189	0.187	0.17	0.166	0.161	0.156	0.164
Raw Data (6(	13 h	0.172	0.51	0.188	0.308	0.185	0.194	0.186	0.163	0.17	0.183	0.183	0.16	0.161	0.155	0.151	0.156
Raw Data (6(	13 h 30 min	0.169	0.536	0.187	0.348	0.184	0.192	0.186	0.16	0.18	0.188	0.188	0.164	0.163	0.155	0.154	0.156
Raw Data (6(	14 h	0.176	0.556	0.191	0.383	0.184	0.215	0.251	0.159	0.174	0.183	0.183	0.16	0.161	0.157	0.154	0.161
Raw Data (6(	14 h 30 min	0.174	0.577	0.19	0.417	0.183	0.209	0.2	0.158	0.175	0.185	0.184	0.161	0.159	0.152	0.15	0.156
Raw Data (6(	15 h	0.174	0.596	0.191	0.459	0.186	0.229	0.212	0.158	0.171	0.186	0.182	0.16	0.162	0.158	0.154	0.162
Raw Data (6(	15 h 30 min	0.175	0.615	0.191	0.49	0.184	0.248	0.23	0.155	0.19	0.19	0.193	0.164	0.164	0.161	0.156	0.164
Raw Data (6(	16 h	0.176	0.635	0.191	0.515	0.184	0.263	0.244	0.155	0.175	0.186	0.185	0.16	0.162	0.156	0.154	0.162
Raw Data (6(	16 h 30 min	0.172	0.652	0.196	0.533	0.188	0.293	0.262	0.16	0.185	0.192	0.191	0.166	0.166	0.158	0.155	0.161
Raw Data (6(	17 h	0.175	0.667	0.196	0.549	0.189	0.336	0.284	0.16	0.178	0.189	0.185	0.162	0.162	0.156	0.153	0.163
Raw Data (6(	17 h 30 min	0.172	0.688	0.2	0.57	0.24	0.465	0.329	0.161	0.183	0.187	0.187	0.162	0.161	0.156	0.152	0.155
Raw Data (6(	18 h	0.173	0.704	0.208	0.586	0.192	0.417	0.364	0.156	0.18	0.189	0.189	0.166	0.164	0.164	0.156	0.162
Raw Data (6(	18 h 30 min	0.174	0.719	0.22	0.603	0.19	0.489	0.412	0.158	0.176	0.188	0.187	0.163	0.162	0.158	0.155	0.161
Raw Data (6(	19 h	0.178	0.736	0.232	0.615	0.191	0.493	0.432	0.157	0.181	0.189	0.188	0.164	0.163	0.158	0.156	0.161
Raw Data (6(	19 h 30 min	0.171	0.751	0.253	0.631	0.196	0.513	0.469	0.159	0.177	0.188	0.185	0.164	0.162	0.157	0.154	0.166
Raw Data (6(	20 h	0.175	0.759	0.279	0.649	0.204	0.531	0.498	0.159	0.175	0.187	0.186	0.164	0.161	0.154	0.154	0.166
Raw Data (6(	20 h 30 min	0.176	0.771	0.294	0.662	0.207	0.544	0.514	0.156	0.182	0.186	0.187	0.162	0.159	0.156	0.155	0.163
Raw Data (6(	21 h	0.172	0.777	0.318	0.671	0.226	0.56	0.527	0.158	0.184	0.197	0.193	0.168	0.166	0.164	0.159	0.167
Raw Data (6(	21 h 30 min	0.172	0.786	0.34	0.688	0.246	0.602	0.548	0.164	0.178	0.191	0.187	0.162	0.161	0.157	0.156	0.166
Raw Data (6(	22 h	0.179	0.795	0.351	0.7	0.268	0.593	0.557	0.16	0.184	0.19	0.191	0.166	0.168	0.165	0.161	0.17
Raw Data (6(	22 h 30 min	0.177	0.804	0.359	0.715	0.282	0.649	0.578	0.161	0.19	0.191	0.19	0.166	0.163	0.161	0.154	0.163
Raw Data (6(	23 h	0.179	0.813	0.372	0.726	0.308	0.624	0.587	0.163	0.184	0.188	0.19	0.164	0.162	0.158	0.155	0.169
Raw Data (6(	23 h 30 min	0.179	0.819	0.38	0.738	0.344	0.64	0.598	0.159	0.185	0.189	0.189	0.163	0.162	0.161	0.157	0.169
Raw Data (6(	24 h	0.171	0.826	0.392	0.744	0.384	0.682	0.616	0.161	0.183	0.192	0.191	0.165	0.163	0.158	0.157	0.17
Raw Data (6(	24 h 30 min	0.185	0.835	0.403	0.753	0.393	0.664	0.622	0.164	0.18	0.188	0.189	0.162	0.164	0.162	0.159	0.17
Raw Data (6(	25 h	0.179	0.839	0.409	0.757	0.412	0.672	0.633	0.16	0.198	0.197	0.197	0.171	0.17	0.165	0.163	0.176
Raw Data (6(	25 h 30 min	0.175	0.841	0.416	0.767	0.432	0.686	0.642	0.161	0.181	0.188	0.192	0.164	0.167	0.163	0.161	0.17
Raw Data (6(	26 h	0.184	0.849	0.423	0.771	0.447	0.699	0.654	0.164	0.189	0.193	0.196	0.167	0.167	0.165	0.157	0.171
Raw Data (6(	26 h 30 min	0.18	0.852	0.432	0.779	0.465	0.71	0.665	0.163	0.185	0.191	0.195	0.162	0.165	0.161	0.156	0.176
Raw Data (6(	27 h	0.176	0.861	0.437	0.783	0.474	0.718	0.674	0.16	0.187	0.19	0.192	0.165	0.165	0.159	0.154	0.17
Raw Data (6(	27 h 30 min	0.181	0.867	0.446	0.791	0.488	0.727	0.688	0.162	0.186	0.19	0.194	0.165	0.165	0.164	0.159	0.176
Raw Data (6(	28 h	0.176	0.872	0.445	0.801	0.496	0.733	0.697	0.162	0.196	0.194	0.204	0.167	0.166	0.163	0.159	0.172
Raw Data (6(	28 h 30 min	0.186	0.877	0.454	0.834	0.502	0.743	0.707	0.161	0.187	0.186	0.194	0.166	0.163	0.16	0.157	0.173
Raw Data (6(	29 h	0.179	0.882	0.46	0.804	0.506	0.752	0.719	0.164	0.188	0.188	0.193	0.162	0.16	0.159	0.155	0.166
Raw Data (6(	29 h 30 min	0.18	0.886	0.461	0.81	0.543	0.782	0.726	0.165	0.19	0.194	0.196	0.165	0.163	0.161	0.158	0.17
Raw Data (6(	30 h	0.184	0.888	0.468	0.816	0.518	0.765	0.733	0.169	0.189	0.188	0.194	0.164	0.163	0.162	0.157	0.175
Raw Data (6(	30 h 30 min	0.181	0.896	0.47	0.822	0.522	0.771	0.732	0.161	0.197	0.193	0.201	0.168	0.169	0.168	0.164	0.181
Raw Data (6(	31 h	0.178	0.9	0.504	0.825	0.546	0.78	0.739	0.164	0.201	0.206	0.204	0.168	0.17	0.163	0.158	0.176
Raw Data (6(	31 h 30 min	0.181	0.9	0.481	0.879	0.63	0.826	0.749	0.159	0.181	0.192	0.195	0.164	0.166	0.163	0.161	0.178
Raw Data (6(	32 h	0.18	0.904	0.486	0.833	0.54	0.794	0.756	0.17	0.192	0.194	0.198	0.169	0.163	0.164	0.161	0.169
Raw Data (6(	32 h 30 min	0.178	0.908	0.485	0.837	0.541	0.803	0.758	0.167	0.204	0.196	0.199	0.165	0.165	0.165	0.16	0.18
Raw Data (6(	33 h	0.186	0.916	0.519	0.852	0.547	0.806	0.766	0.173	0.192	0.192	0.196	0.164	0.164	0.166	0.162	0.18
Raw Data (6(	33 h 30 min	0.184	0.95	0.542	0.852	0.552	0.815	0.771	0.167	0.198	0.195	0.203	0.173	0.17	0.17	0.167	0.184
Raw Data (6(	34 h	0.18	0.919	0.499	0.854	0.571	0.85	0.795	0.172	0.198	0.202	0.201	0.169	0.166	0.161	0.156	0.179
Raw Data (6(	34 h 30 min	0.184	0.938	0.525	0.857	0.559	0.825	0.78	0.168	0.19	0.194	0.197	0.166	0.168	0.164	0.16	0.183
Raw Data (6(	35 h	0.188	0.931	0.509	0.864	0.563	0.829	0.786	0.166	0.2	0.199	0.202	0.168	0.167	0.164	0.158	0.182
Raw Data (6(	35 h 30 min	0.183	0.954	0.511	0.865	0.596	0.833	0.794	0.167	0.194	0.195	0.201	0.167	0.166	0.164	0.164	0.178
Raw Data (6(	36 h	0.185	0.933	0.518	0.868	0.574	0.843	0.798	0.171	0.197	0.195	0.199	0.165	0.167	0.165	0.158	0.175
Raw Data (6(	36 h 30 min	0.187	0.936	0.52	0.873	0.579	0.851	0.805	0.168	0.2	0.193	0.196	0.167	0.166	0.164	0.16	0.18
Raw Data (6(	37 h	0.184	0.938	0.525	0.876	0.584	0.854	0.81	0.167	0.192	0.197	0.197	0.165	0.166	0.163	0.16	0.189
Raw Data (6(	37 h 30 min	0.182	0.938	0.527	0.877	0.587	0.855	0.81	0.168	0.201	0.202	0.212	0.173	0.173	0.173	0.166	0.19
Raw Data (6(	38 h	0.187	0.981	0.592	0.885	0.591	0.864	0.82	0.168	0.205	0.205	0.202	0.172	0.166	0.162	0.159	0.185
Raw Data (6(	38 h 30 min	0.18	0.945	0.535	0.885	0.596	0.86	0.824	0.174	0.202	0.197	0.205	0.175	0.168	0.167	0.161	0.185
Raw Data (6(	39 h	0.183	0.947	0.539	0.888	0.603	0.868	0.821	0.17	0.212	0.199	0.206	0.173	0.171	0.172	0.168	0.19
Raw Data (6(	39 h 30 min	0.187	0.951	0.543	0.895	0.604	0.874	0.825	0.169	0.198	0.192	0.198	0.166	0.167	0.165	0.162	0.186
Raw Data (6(	40 h	0.191	0.948	0.547	0.896	0.609	0.879	0.835	0.168	0.216	0.19	0.198	0.168	0.168	0.171	0.163	0.191
Raw Data (6(	40 h 30 min	0.189	0.948	0.548	0.898	0.612	0.88	0.835	0.171	0.199	0.194	0.2	0.167	0.168	0.167	0.164	0.19
Raw Data (6(	41 h	0.186	0.951	0.552	0.9	0.616	0.882	0.842	0.17	0.205	0.199	0.2	0.166	0.169	0.162	0.163	0.186
Raw Data (6(	41 h 30 min	0.178	0.991	0.591	0.92	0.622	0.886	0.836	0.173	0.21	0.198	0.201	0.171	0.173	0.17	0.168	0.193
Raw Data (6(	42 h	0.182	0.953	0.56	0.908	0.624	0.892	0.847	0.184	0.208	0.196	0.199	0.17	0.171	0.17	0.168	0.196
Raw Data (6(	42 h 30 min	0.19	0.97	0.574	0.906	0.63	0.896	0.846	0.18	0.21	0.194	0.2	0.17	0.173	0.167	0.164	0.197
Raw Data (6(	43 h	0.19	0.955	0.569	0.911	0.633	0.898	0.854	0.17	0.209	0.196	0.203	0.172	0.173	0.169	0.166	0.195
Raw Data (6(	43 h 30 min	0.18	0.958	0.57	0.907	0.638	0.901	0.851	0.18	0.209	0.201	0.204	0.17	0.171	0.165	0.162	0.189
Raw Data (6(	44 h	0.188	0.964	0.575	0.913	0.68	0.912	0.855	0.172	0.212	0.195	0.203	0.169	0.17	0.168	0.166	0.192
Raw Data (6(	44 h 30 min	0.192	0.961	0.578	0.915	0.657	0.995	0.856	0.17	0.213	0.195	0.202	0.17	0.168	0.17	0.166	0.193
Raw Data (6(	45 h	0.184	0.963	0.593	0.922	0.642	0.903	0.858	0.17	0.207	0.198	0.201	0.169	0.168	0.167	0.164	0.196
Raw Data (6(	45 h 30 min	0.189	0.966	0.585	0.916	0.644	0.906	0.86	0.169	0.208	0.198	0.202	0.168	0.169	0.168	0.163	0.194
Raw Data (6(	46 h	0.191	0.967	0.588	0.919	0.663	0.912	0.863	0.172	0.223	0.2	0.206	0.175	0.172	0.17	0.162	0.202
Raw Data (6(	46 h 30 min	0.194	0.965	0.593	0.921	0.649	0.912	0.866	0.176	0.212	0.198	0.203	0.171	0.169	0.168	0.165	0.201
Raw Data (6(	47 h	0.188	0.964	0.592	0.917	0.656	0.914	0.867	0.179	0.208	0.199	0.204	0.173	0.172	0.171	0.168	0.196
Raw Data (6(	47 h 30 min	0.199	0.997	0.631	0.927	0.668	0.919	0.87	0.174	0.21	0.197	0.204	0.172	0.172	0.173	0.168	0.201
Raw Data (6(	48 h	0.184	0.97	0.601	0.937	0.658	0.92	0.873	0.181	0.214	0.201	0.201	0.173	0.166	0.167	0.163	0.191
Raw Data (6(	48 h 30 min	0.185	0.969	0.604	0.929	0.662	0.956	0.877	0.18	0.212	0.197	0.203	0.168	0.168	0.168	0.164	0.195
Raw Data (6(	49 h	0.189	0.97	0.606	0.927	0.664	0.924	0.877	0.174	0.213	0.2	0.206	0.176	0.171	0.17	0.165	0.199
Raw Data (6(	49 h 30 min	0.19	0.973	0.616	0.93	0.669	1.002	0.915	0.178	0.229	0.201	0.206	0.178	0.178	0.177	0.169	0.205
Raw Data (6(	50 h	0.187	0.974	0.612	0.931	0.669	0.925	0.88	0.175	0.217	0.202	0.203	0.173	0.168	0.172	0.165	0.204
Raw Data (6(	50 h 30 min	0.185	0.97	0.613	0.926	0.668	0.925	0.879	0.172	0.224	0.204	0.203	0.173	0.169	0.17	0.167	0.205
Raw Data (6(	51 h	0.192	0.973	0.615	0.928	0.67	0.926	0.881	0.169	0.226	0.211	0.21	0.178	0.176	0.178	0.171	0.21
Raw Data (6(	51 h 30 min	0.193	0.974	0.616	0.93	0.67	0.93	0.883	0.177	0.224	0.201	0.204	0.172	0.17	0.17	0.165	0.201
Raw Data (6(	52 h	0.199	0.973	0.618	0.95	0.677	0.931	0.884	0.18	0.234	0.203	0.204	0.174	0.174	0.173	0.167	0.209
Raw Data (6(	52 h 30 min	0.196	0.972	0.618	0.929	0.668	0.928	0.882	0.17	0.222	0.202	0.204	0.172	0.171	0.172	0.167	0.205
Raw Data (6(	53 h	0.188	0.973	0.621	0.932	0.669	0.932	0.886	0.182	0.222	0.202	0.202	0.171	0.171	0.169	0.16	0.195
Raw Data (6(	53 h 30 min	0.197	0.972	0.621	0.929	0.67	0.929	0.886	0.171	0.233	0.203	0.203	0.171	0.172	0.168	0.165	0.204
Raw Data (6(	54 h	0.199	0.974	0.624	0.935	0.678	0.932	0.89	0.182	0.224	0.201	0.205	0.171	0.171	0.17	0.166	0.206
Raw Data (6(	54 h 30 min	0.193	0.97	0.625	0.93	0.674	0.931	0.889	0.186	0.222	0.2	0.205	0.175	0.177	0.174	0.17	0.21
Raw Data (6(	55 h	0.188	0.969	0.628	0.93	0.678	0.932	0.889	0.178	0.227	0.205	0.205	0.171	0.165	0.167	0.163	0.212
Raw Data (6(	55 h 30 min	0.196	0.97	0.628	0.93	0.675	0.933	0.891	0.167	0.228	0.204	0.21	0.175	0.181	0.176	0.17	0.22
Raw Data (6(	56 h	0.197	0.971	0.631	0.932	0.684	0.937	0.894	0.182	0.226	0.203	0.202	0.173	0.169	0.171	0.166	0.208
Raw Data (6(	56 h 30 min	0.197	0.971	0.628	0.929	0.677	0.934	0.891	0.172	0.235	0.204	0.209	0.174	0.175	0.169	0.169	0.213
Raw Data (6(	57 h	0.201	0.972	0.633	0.932	0.679	0.935	0.893	0.175	0.236	0.204	0.204	0.174	0.173	0.172	0.169	0.213
Raw Data (6(	57 h 30 min	0.202	0.97	0.634	0.935	0.679	0.939	0.897	0.189	0.239	0.201	0.207	0.176	0.176	0.171	0.168	0.217
Raw Data (6(	58 h	0.191	0.97	0.633	0.933	0.678	0.938	0.895	0.182	0.242	0.204	0.21	0.179	0.173	0.173	0.17	0.217
Raw Data (6(	58 h 30 min	0.193	0.968	0.635	0.931	0.68	0.938	0.897	0.18	0.238	0.207	0.206	0.184	0.181	0.177	0.172	0.224
Raw Data (6(	59 h	0.199	0.972	0.638	0.936	0.679	0.941	0.899	0.189	0.26	0.209	0.214	0.185	0.187	0.182	0.18	0.22
Raw Data (6(	59 h 30 min	0.196	0.969	0.634	0.932	0.677	0.939	0.895	0.173	0.255	0.213	0.219	0.186	0.185	0.18	0.178	0.227
Raw Data (6(	60 h	0.192	0.968	0.637	0.934	0.68	0.94	0.899	0.182	0.242	0.204	0.214	0.179	0.174	0.171	0.17	0.215
Raw Data (6(	60 h 30 min	0.199	0.968	0.636	0.933	0.678	0.94	0.899	0.179	0.249	0.214	0.213	0.188	0.184	0.18	0.173	0.224
Raw Data (6(	61 h	0.202	0.966	0.639	0.935	0.68	0.94	0.898	0.176	0.242	0.21	0.205	0.178	0.183	0.174	0.176	0.226
Raw Data (6(	61 h 30 min	0.202	0.971	0.639	0.933	0.681	0.941	0.898	0.176	0.235	0.203	0.211	0.178	0.18	0.175	0.172	0.224
Raw Data (6(	62 h	0.208	0.968	0.641	0.931	0.683	0.94	0.896	0.18	0.25	0.206	0.212	0.183	0.183	0.177	0.175	0.222
Raw Data (6(	62 h 30 min	0.202	0.968	0.641	0.931	0.68	0.94	0.899	0.176	0.268	0.219	0.221	0.194	0.189	0.187	0.184	0.238
Raw Data (6(	63 h	0.202	0.971	0.642	0.933	0.678	0.942	0.901	0.178	0.261	0.208	0.213	0.181	0.179	0.174	0.172	0.23
Raw Data (6(	63 h 30 min	0.195	0.968	0.643	0.933	0.681	0.942	0.903	0.181	0.256	0.209	0.207	0.199	0.18	0.181	0.178	0.24
Raw Data (6(	64 h	0.205	0.97	0.644	0.932	0.678	0.943	0.902	0.186	0.259	0.208	0.21	0.181	0.179	0.175	0.172	0.228
Raw Data (6(	64 h 30 min	0.2	0.965	0.644	0.931	0.68	0.941	0.904	0.195	0.257	0.215	0.213	0.19	0.177	0.181	0.175	0.236
Raw Data (6(	65 h	0.203	0.968	0.645	0.933	0.676	0.942	0.902	0.186	0.258	0.208	0.213	0.185	0.181	0.183	0.181	0.232
Raw Data (6(	65 h 30 min	0.207	0.967	0.645	0.934	0.68	0.945	0.905	0.191	0.252	0.212	0.21	0.181	0.179	0.172	0.173	0.221
Raw Data (6(	66 h	0.205	0.967	0.647	0.935	0.68	0.943	0.903	0.183	0.251	0.211	0.209	0.184	0.186	0.181	0.179	0.235
Raw Data (6(	66 h 30 min	0.205	0.967	0.644	0.932	0.678	0.943	0.903	0.183	0.262	0.214	0.216	0.192	0.178	0.18	0.18	0.234
